# Intraradical colonization by arbuscular mycorrhizal fungi triggers induction of a lipochitooligosaccharide receptor

**DOI:** 10.1038/srep29733

**Published:** 2016-07-20

**Authors:** S. R. Rasmussen, W. Füchtbauer, M. Novero, V. Volpe, N. Malkov, A. Genre, P. Bonfante, J. Stougaard, S. Radutoiu

**Affiliations:** 1 Department of Molecular Biology and Genetics, Centre for Carbohydrate Recognition and Signalling, Aarhus University, Denmark; 2 Department of Life Science and Systems Biology, University of Torino, Italy

## Abstract

Functional divergence of paralogs following gene duplication is one of the mechanisms leading to evolution of novel pathways and traits. Here we show that divergence of *Lys11* and *Nfr5* LysM receptor kinase paralogs of *Lotus japonicus* has affected their specificity for lipochitooligosaccharides (LCOs) decorations, while the innate capacity to recognize and induce a downstream signalling after perception of rhizobial LCOs (Nod factors) was maintained. Regardless of this conserved ability, *Lys11* was found neither expressed, nor essential during nitrogen-fixing symbiosis, providing an explanation for the determinant role of *Nfr5* gene during *Lotus*-rhizobia interaction. *Lys11* was expressed in root cortex cells associated with intraradical colonizing arbuscular mycorrhizal fungi. Detailed analyses of *lys11* single and *nfr1nfr5lys11* triple mutants revealed a functional arbuscular mycorrhizal symbiosis, indicating that *Lys11* alone, or its possible shared function with the Nod factor receptors is not essential for the presymbiotic phases of AM symbiosis. Hence, both subfunctionalization and specialization appear to have shaped the function of these paralogs where *Lys11* acts as an AM-inducible gene, possibly to fine-tune later stages of this interaction.

The association of plants with arbuscular mycorrhiza (AM) is estimated to be as old as land plants themselves^1^,^2^. Furthermore, development of elaborate root systems associated with the move to the dryer soil environment is thought to have enhanced plant stability, water and nutrient acquisition but also to have improved the accommodation of mycorrhizal fungi^3^,^4^. As a consequence, different types of roots with specific anatomy and/or ability to accommodate the symbiotic fungus have evolved among plant lineages^5^,^6^. Genetic and biochemical studies have recently identified central components involved in the molecular cross-talk between plant and AM fungi that mediate the symbiotic accommodation of the microbe. Plant-secreted strigolactones stimulate hyphal branching^7^,^8^ and secretion of specific molecules, the Myc factors, is initiated. Two types of chitin-derived molecules have been identified as Myc factors: short-chain chitin (CO4-CO5)^9^ and lipochitooligosaccharides (LCOs) with a variety of side-chain decorations^10^. Perception of these molecules by plant cells leads to a signal cascade that reprograms the root for symbiotic association^11^,^12^,^13^. Presymbiotic signalling at the root epidermis is followed by the activation of a second generation of signalling events when intraradical fungal hyphae colonize the root cortex and the periarbuscular membrane is formed in the inner cortical cells^14^,^15^. Our current understanding of the signalling between the intraradical fungus and host is still in its infancy due to the complex genetic make-up of the fungus, its obligate biotrophic nature and asynchronous infection^16^. On the other hand, a more in-depth understanding of the symbiotic microbe-host signalling has been obtained from studies of the nitrogen-fixing symbiosis with rhizobia. Evolution of this bacterial endosymbiosis in legumes appears to have recruited part of the molecular hardware required for AM accommodation inside host roots^17^,^18^. The existence of a common genetic program for the two symbioses was revealed by legume genetics, and subsequent genetic complementation experiments where corresponding monocot components performed the same function during AM symbiosis in legumes^17^,^18^. A leucine rich repeat (LRR) receptor kinase SYMRK integrates the signal transduction initiated by the symbiotic fungus and/or bacteria leading to perinuclear calcium oscillations^19^,^20^. These are decoded by the calcium- and calmodulin dependent protein kinase, CCaMK^21^,^22^ that activates specific transcription factors whose assortment ultimately results in development of nitrogen-fixing nodules or colonisation by mycorrhizal fungi, respectively^23^,^24^. It became clear that this common genetic program of the host is an extension of a common strategy for microbial signal recognition, where very similar types of host receptors might be involved. Nitrogen-fixing rhizobia produce Nod-factors, whose structure resemble the acylated- forms of Myc factors, but vary with respect to their side-chain decorations^10^,^25^,^26^. Interestingly, when individually tested, short chitin oligomers and their derivatives are all able to induce calcium spiking in root epidermal cells^9^,^27^, or to activate molecular responses that can overlap with each other or with those induced by the Nod factor^11^,^14^,^28^. During root nodule symbiosis (RNS), specific recognition of the Nod-factors by LysM receptor kinases, NFR1 and NFR5 in *Lotus*, links rhizobial signalling to SYMRK and leads to activation of the common symbiotic pathway in the root^28^,^29^,^30^. Mutation of *Nfr1* or *Nfr5* results in complete block of nitrogen-fixing symbiosis^28^,^29^. However, the role of the individual chitin-type signals in AM symbiosis remained so far difficult to decipher, and the recognition of presymbiotic Myc factor(s) upstream of SYMRK is less clear. Studies based on phenotypic analyses of the chitin receptor mutant (*cerk1*) in rice, and of Nod factor receptor mutant *nfr1* in *Lotus* showed delayed or reduced root colonization, suggesting a possible role of COs and/or LCOs signalling in the initial steps of the fungal-host interaction^31^,^32^. In tomato and *Parasponia*, transcript downregulation of presumed NFR5-type receptors led to either reduced colonisation, or reduced arbuscule formation, respectively^33^,^34^. Nevertheless, well-defined plant mutants blocked in presymbiotic or cortical chitin/LCO perception upstream of SYMRK have not been found, and therefore receptor(s) of the Myc factor(s) remain to be unequivocally identified. The family of LysM receptor kinase has enlarged in legumes compared to other plant species^35^,^36^,^37^. A tempting hypothesis for this evolutionary expansion in legumes is to ensure unique identification, and concomitant accommodation of, or defense against a diverse panel of microbes. Tandem or segmental genome duplications resulted in multiple *Nfr1*-type receptors (four in *Lotus, Lys1, Lys2, Lys6, Lys7* and seven in *Medicago, Lyk1, Lyk2, Lyk4, Lyk6, Lyk7, Lyk8, Lyk9*) that all encode proteins with characteristic subdomains of active kinases^35^,^37^. The *Nfr5*, on the other hand, has a paralogue encoding a protein with similar (pseudo)kinase domains, *Lys11* in *Lotus*^37^ or *Lyr1* in *Medicago*^35^ that have been suggested as receptors of AM- derived LCO in legumes^38^.

In this study we investigate the regulation, signalling capacities and involvement of *Lys11* in root symbioses with nitrogen-fixing rhizobia and arbuscular mycorrhizal fungi in *Lotus japonicus*. We show that LYS11 can perceive LCOs and initiate intracellular signalling, but has different requirements for LCO decorations and a different expression pattern compared to *Nfr5*. Our detailed phenotypic analysis based on single or combinatorial mutant plants revealed on the other hand, no obvious defects during RNS or AM symbioses, indicating that a more intricate signalling exists in legumes during AM symbiosis, than previously inferred.

## Results

### LYS11 complements the symbiotic function of NFR5

**Figure 1:**
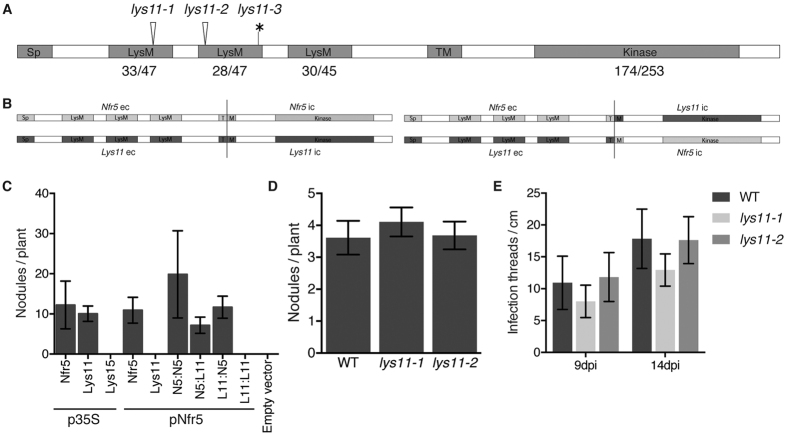
*Lys11* can complement *Nfr5* but is not required for root nodule symbiosis. (**A**) Domain structure and location of mutations on LYS11. *lys11*-*1* and *lys11*-*2* are LORE1 insertion mutants in Gifu at C268, or T338, respectively (arrow head). *lys11*-*3* is a TILLING mutation (G411 to A) in MG20, causing a premature stop codon (*). The predicted domains signal peptide (Sp), LysM domains (LysM), transmembrane (TM), and kinase are shown. The number of identical amino acids in LYS11 and NFR5 for LysM and kinase domains is indicated relative to the number of amino acids in the respective domain. (**B**) Schematic representation of the *Nfr5*–*Lys11* swap constructs where extracellular (ec) and intracellular (ic) regions are combined, and the corresponding controls obtained by using Golden Gate cloning strategy (Methods). (**C**) Average number of nodules formed by the *nfr5*-*2* mutant plants when transformed with full length *Nfr5, Lys11* or *Nfr5* – *Lys11* swap constructs after inoculation with *M. loti*. These constructs were driven by 35S (*p35S*) or *Nfr5 (pNfr5*) promoters. (**D**) Average number of nodules formed by *lys11*-*1, lys11*-*2* and Gifu wild-type plants after five weeks of inoculation with *M. loti*. (**E**) Average number of infection threads formed by *lys11*-*1, lys11*-*2* and Gifu wild-type plants at 9 and 14 days after inoculation with *M. loti*. Error bars show the 95% confidence interval.

Among LysM receptor kinase proteins encoded by *Lotus japonicus* genome, LYS11 shares the highest level of identity (66%), and has a similar domain structure to NFR5 (Fig. 1A, Supplementary Fig. S1)^37^. Therefore, a diverged ability to perceive LCO, or to initiate an intracellular signalling for these receptors could explain the lack of functional redundancy with NFR5 during RNS^28^. To test this hypothesis, we analysed the ability of LYS11 to functionally complement the previously described function and molecular features of NFR5.

First, we investigated the cellular localization of LYS11 and found that the YFP tagged version of LYS11 localizes to the plasma membrane in *N. benthamiana* leaf cells (Supplementary Fig. S2A,B). Next, we used the same heterologous system and bimolecular fluorescence complementation to assess the capacity of LYS11 to interact with NFR1. Co-infiltration of *Lys11* tagged with the N-terminal region of YFP together with the kinase inactive (T428A) version of *Nfr1* tagged with the C-terminal region of YFP led to a fully restored YFP signal at the plasma membrane, indicating co-localisation and heterodimer complex formation, as observed with NFR5^39^ (Supplementary Fig. S2C,D). Finally, we tested if LYS11 can form functional heterodimers with NFR1 that activate a signalling cascade leading to cell death^39^ when co-expressed in *N. benthamiana* leaves. We observed that co-expression of *Lys11* with the kinase active *Nfr1*, but not with *Nfr5*, led to cell death activation in the infiltrated leaves, indicative of a functional NFR1-LYS11 signalling complex (Supplementary Fig. S2E,F).

Next, we asked whether *Lys11* could complement the function of *Nfr5* during root nodule symbiosis^28^. For this we produced various synthetic constructs where the constitutive 35S-CaMV, or *Nfr5* promoter drove the expression of *Lys11* (Fig. 1B). These constructs were introduced in the *nfr5*-2 mutant via *Agrobacterium rhizogenes*, and transgenic roots were screened for their ability to develop root nodules after *Mesorhizobium loti* inoculation. We observed that constitutive expression of *Lys11* from the 35S promoter led to full complementation of *nfr5*-*2* symbiotic phenotype (Fig. 1C, Supplementary Fig. S3). Functional nodules formed on the transformed mutant roots after *M. loti* inoculation (Supplementary Fig. S3). The number and the appearance of the nodules formed on *Lys11* transformed roots were similar to the ones formed on the *Nfr5* overexpressing roots. By contrast, no complementation was observed when *Lys11* was expressed under the control of *Nfr5* promoter indicating that LYS11 protein has a reduced affinity for Nod factors or a lower intracellular signalling activity compared to NFR5 (Fig. 1C, Supplementary Fig. S3). To investigate these two scenarios, we created two synthetic chimeric constructs (LYS11-NFR5 and NFR5-Lys11) where the intracellular regions were swapped between these two receptor proteins (Fig. 1B). We assayed the complementation capacities of these chimeric constructs when expressed under the control of *Nfr5* promoter. Analysis of the transformed roots showed that both chimeric constructs driven by *Nfr5* promoter were able to rescue the *nfr5*-*2* with comparable efficiencies (Fig. 1C). This shows that the extracellular or intracellular domains of LYS11 enable efficient signaling when combined with the corresponding domains of NFR5. However, when together an enhancement of their signaling capacity from the 35S promoter is needed for efficient functional complementation. In order to find whether this ability to complement the symbiotic defective phenotype is a general characteristic for the NFR5-type proteins when overexpressed, we tested whether *Lys15*^37^, which phylogenetically is the closest receptor protein of NFR5-LYS11 pair, would also complement the *nfr5*-*2* mutant when controlled by the 35S promoter. The *nfr5*-*2* plants expressing *p35S*:*Lys15* construct retained the nitrogen-starved phenotype, and no nodules were observed after 5 weeks in the presence of *M. loti* (Fig. 1C, Supplementary Fig. S3).

These results show that both LYS11 and NFR5 can perceive lipochitooligosaccharides and initiate an intracellular signalling cascade that results in functional root nodule formation after inoculation with Nod-factor producing rhizobia.

### *Nfr5* and *Lys11* have distinct expression patterns and roles during root nodule symbiosis

Our results from complementation analyses revealed that *Lys11* could complement the symbiotic defective phenotype of *nfr5* mutant. This competence was dependent on *Lys11* expression level/pattern. This observation, combined with the null phenotype of *nfr5*-*2* mutant in the presence of rhizobia, and the lack of *Lys11* transcript regulation during root nodule symbiosis^38^ prompted us to investigate the spatio-temporal expression of *Lys11*. For this, we monitored the activity of *Lys11* promoter using a *pLys11*:*GUS* transcriptional reporter (1.5 kb promoter) in transformed *Lotus* roots. We observed that unlike *Nfr5* promoter that was active in uninoculated roots (Kawaharada *et al*., submitted), the activity of *Lys11* promoter was below visual detection in similar root cells (Supplementary Fig. S4). Treatment with *M. loti* purified Nod factors (24 h), or inoculation with *M. loti* did not lead to activation of *Lys11* promoter inside roots or nodules at any developmental stage (Supplementary Fig. S4B,C, Supplementary Table S1). These observations confirmed the previous results obtained from transcript level measurement based on qRT-PCR^37^, and provide a clear explanation for the lack of redundancy between *Nfr5* and *Lys11* during rhizobial symbiosis.

To further resolve the potential function of *Lys11* during root-nodule symbiosis we identified three independent homozygous mutant lines with nonsense mutations or LORE1 retroelement insertions from *Lotus* mutant collections^40^ (Fig. 1A). These mutants were investigated for their ability to develop root nodules and sustain efficient nitrogen-fixing symbiosis. Assayed on plates or in greenhouse conditions wild-type and mutant plants revealed similar capacities to sustain RNS. A comparable number of nodules, infection threads, and symbiotic competency illustrated by the shoot length and appearance revealed that *lys11* mutants resemble wild-type plants after inoculation with *M. loti* (Fig. 1D,E, Supplementary Fig. S5).

Together, our results from mutant and expression pattern analyses show that *Lys11*, regardless of its ability to perceive Nod factors, does not seem to play a role in rhizobial symbiosis.

### *Lys11* transcriptional activation depends on internal accommodation of AM fungi

**Figure 2:**
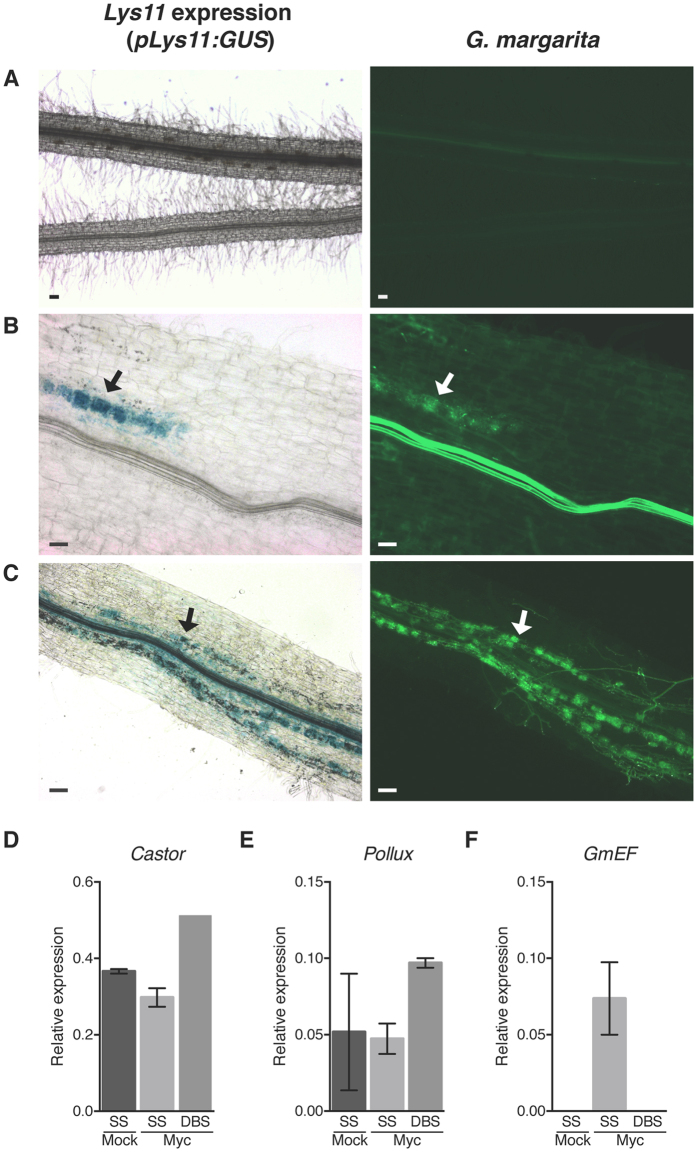
An internal AM fungal signal is required for activation of *Lys11* promoter. Wild-type roots expressing *pLys11*:GUS were non-inoculated (Mock) or *G. margarita*-inoculated (Myc) using the single sandwich (SS) or the double sandwich growth systems (DBS). (**A**) Mock-treated roots and Myc-inoculated roots grown in the DBS system showed no activity of *Lys11* promoter and where not infected by the fungus. Myc-inoculated roots grown in the SS system showed *Lys11* promoter activity after 7 (**B**) and 28 days (**C**), and promoter activity correlated with mycorrized areas of the root (arrows in (**B**,**C**)). Increased expression of *Castor* (**D**) and *Pollux* (**E**) in *Lotus* roots grown in the DBS system shows that fungal signals are perceived by the host, but fungal mass accumulation visualised by *G. margarita* EF (elongation factor) expression inside roots (**F**) is only detected in the SS system. *Lys11* promoter activation is visualised in blue as a measure of GUS activity (black arrow) and fungal structures are visualised in green based on fluorescence detection after WGA Alexa Fluor 488 staining (AM fungus- white arrow). Scale bars in (**A**–**C**) represent 50 μm. Error bars (**D**–**F**) show the standard deviation.

The absence of gene expression and undetectable function during root-nodule symbiosis can be explained if *Lys11* is a pseudogene. To examine this possibility we investigated the regulation of *Lys11* expression during symbiosis with arbuscular mycorrhizal fungi that produce LCOs, in addition to short- (CO4-CO5) and long-chain (CO8) chitin molecules. Previous analyses of *Lys* genes regulation after CO8 treatment did not reveal a significant change in *Lys11* transcript levels^37^. Individual and combinations of AM-specific elicitors and derivatives of these were therefore tested for *Lys11* promoter activation. First, we treated transgenic roots expressing *pLys11*:GUS with mono-solutions of different chitin-oligomers (CO2–5) and analysed the reporter gene activity after 24 hours. Like in the case of Nod factor treatment, no activation of *Lys11* promoter was observed in roots exposed to any of these treatments (Supplementary Table S1). Next, we made use of the single- and double sandwich systems described previously^41^ in order to fully explore the signalling capacity of AM fungi, which probably use a more complex molecular signalling than the identified COs and LCOs^42^. The roots were exposed to either direct contact with *Gigaspora margarita* (single sandwich), or to fungal-host exchanged signalling molecules only (double sandwich). Histochemical analyses of the *pLys11*:*GUS* transformed roots from the two systems showed that *Lys11* promoter is active, but only in transgenic roots interacting directly with *Gigaspora margarita* that developed intraradical symbiotic structures (Fig. 2A–C, Supplementary Table S1). To further ensure that the roots lacking expression of *pLys11*:*GUS* (double sandwich) were indeed perceiving presymbiotic fungal signals we examined the transgenic roots for induced expression of *Castor* and *Pollux*, two markers for early AM signalling initiation in the host^41^. Our results obtained from qRT-PCR analyses revealed that both *Castor* and *Pollux* had higher level of expression in the double sandwich system (Fig. 2D–F), indicating that AM signalling molecules were perceived by the *pLys11*:*GUS* transgenic roots, but were insufficient to induce *Lys11* promoter activation (Fig. 2A).

**Figure 3:**
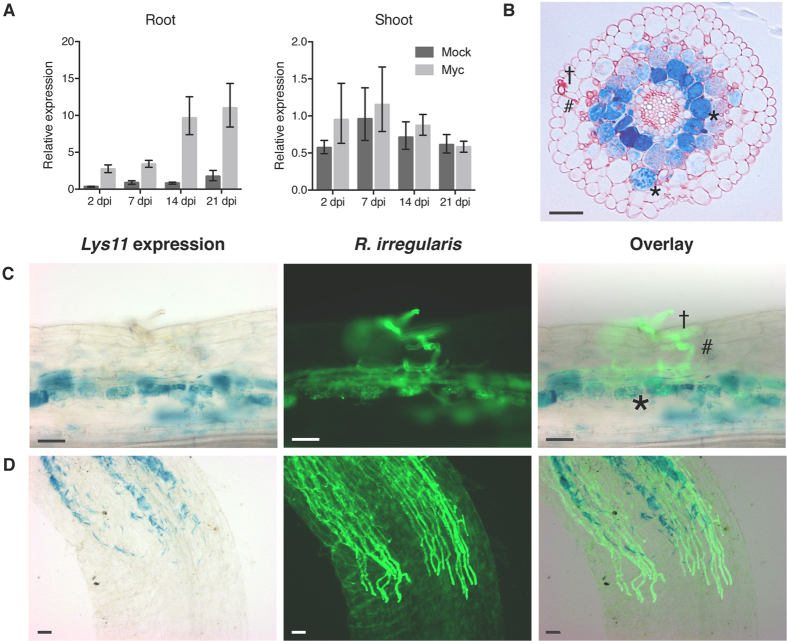
*Lys11* is upregulated in inner cortical root cells of mycorrhized areas. (**A**) *Lys11* expression is induced in the roots but not in the shoots of arbuscular mycorrhiza inoculated plants. (**B**) Thin section of the root region expressing *Lys11* shows specific promoter activity in the inner cortical cells visualised in blue after staining for GUS activity. (**C**,**D**) Whole root concomitant visualisation of GUS activity (blue staining) and fungal structures (green fluorescence after WGA staining) shows *Lys11* promoter activity in mycorrhized areas of the inner cortex, which include inner cortical cells with arbuscules or in close proximity to mycorrhizal hyphae (*), and no promoter activity in the epidermal (†) or outer cortical cells (#) even when in contact with the fungal hyphae. *Lys11* expression follows intraradical fungal infection (**C**) Scale bars (**B**–**D**) represent 50 μm, error bars in (**A**) show the 95% confidence interval.

This specific regulation of *Lys11* led us to investigate in detail the spatio-temporal regulation of *Lys11* promoter activity in AM inoculated roots. We observed a progressive increase in *Lys11* promoter activity revealed by the increasing root zones and intensity of GUS staining following the time-dependent AM colonization (14 and 21 dpi) (Supplementary Fig. S6). These data were supported by transcript quantification of *Lys11* in roots and shoots that revealed an AM-regulated expression confined to below-ground organs (Fig. 3A). Inspection of microtome transversal sections obtained from roots expressing *pLys11*:*GUS* revealed a specific promoter activity restricted to the inner cortical cells (Fig. 3B). Concomitant visualisation of the transcriptional reporter and AM fungal structures showed *Lys11* promoter activity in cells with arbuscules, in the inner cortical cells found in close proximity to intraradical hyphae (Fig. 3C), and behind the growth front of the fungal hyphae (Fig. 3D). Epidermal and outer cortical cells showed no promoter activity even when in contact with the fungal hyphae (Fig. 3A,C). To further resolve these observations genetically we took advantage of this transcriptional reporter and investigated *Lys11* activation in different mutant background where the Nod factor signalling or common symbiotic signalling pathways were impaired. We observed that Nod factor perception or *Nsp2*- dependent transcriptional activation are not required for *Lys11* activation since *nfr1*-*1, nfr5*-*2, nfr1*-*1nfr5*-*2*, and *nsp2*-*3* mutants had a similar pattern and intensity of *Lys11* promoter as in wild-type (Supplementary S7A–D). On the other hand, *symRK*-*2* and *ccamK*-*3* mutants that were impaired in AM colonization and arbuscule formation, failed to induce *Lys11* promoter activation (Supplementary Fig. S7E,F). These observations confirmed our results from *G. margarita* inoculation (Fig. 2) that an intraradical signalling produced as a consequence of fungal accommodation is the main driver for *Lys11* activation in the inner cortical cells.

Together, these results show that *Lys11* is not a pseudogene, but is transcriptionally regulated during intraradical accommodation of AM fungus, and downstream of symbiotic genes controlling the early onset of the symbiosis.

### Lys11 mutants form efficient symbiosis with AM fungi

**Figure 4:**
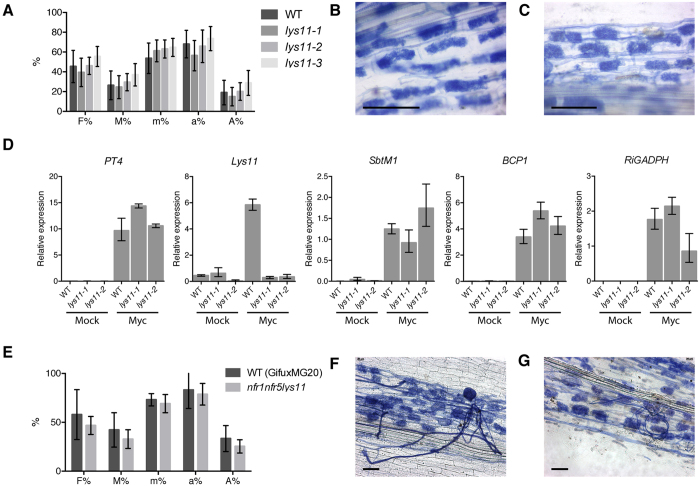
Arbuscular mycorrhizal fungi colonize efficiently *lys11* and *nfr1nfr5lys11*. (**A**) Wild-type (WT), *lys11*-*1, lys11*-*2* and *lys11*-*3* roots show similar degree of fungal colonization and arbuscule formation. Arbuscules formed in WT (**B**) and *lys11* mutants ((**C**) shows *lys11*-*1* as a representative) were morphologically similar. (**D**) Transcript levels of host (*PT4, SbtM1, BCP1*) and fungus (*RiGAPDH*) marker genes in non-inoculated (Mock) or *R. irregularis*-inoculated (Myc) roots are similar in WT and *lys11* mutants. *Lys11* was induced in WT samples but not in *lys11* mutants. (**E**) Wild-type (bred from Gifu and MG20 cross) and *nfr1nfr5lys11* roots show similar degree of fungal colonization and arbuscule formation. Arbuscules formed in WT (**F**) and *nfr1nfr5lys11* mutants (**G**) were morphologically similar. *R. irregularis* inoculum was used and plants were grown in system II- Methods. F%, M%, m%, a% and A% are defined in Methods. Scale bars (**B**,**C**,**F**,**G**) represent 50 μm. Error bars show 95% confidence interval in (**A**,**D**,**E**).

Our analyses revealed a specific spatio-temporal activation of *Lys11* in the cells with arbuscules, or in their close vicinity. In order to better understand the signalling function of LYS11 during AM symbiosis we asked whether LYS11 is required for arbuscule formation. To test this we analysed *lys11* mutants for their ability to sustain efficient arbuscule formation and root symbioses with AM fungi. Wild-type and *lys11* mutants were inoculated with *R. irregularis* and the whole root material was analysed for efficient fungal colonization four weeks after inoculation (System II: see material and method). We observed that *lys11* mutants and wild-type had a similar level of fungal colonisation, and a similar frequency of cortical cells with arbuscules (Fig. 4A). Detailed microscopic analyses of the arbuscules formed in mutant and wild-type roots revealed no obvious differences (Fig. 4B,C, Supplementary Fig. S8A,B). Based on the knowledge that AM symbiosis establishment is both inoculum, status or density dependent we further investigated the ability of *lys11* mutants to establish AM symbiosis in the presence of a different *R. irregularis* inoculum or in the presence of a different AM fungus, *G. margarita*. The second *R. irregularis* inoculum was obtained from a different provider and was supplied in a different formulation (System III: see material and method) and therefore the amount of inoculum (spore and mycelia) was different. In the case of *G. margarita*, individually collected spores were used as inoculum. For all types of inoculum, quantification of the root fraction colonized by the AM fungi, or the arbusculated cortical cells revealed similar frequencies for *lys11* mutants and wild-type roots (Supplementary Fig. S8D,E). Furthermore, no obvious morphological differences between arbuscules in mutant and wild-type were detected (Supplementary Fig. S8A,B). These results obtained from three different inoculation methodologies indicate that AM fungi are able to establish themselves equally well in roots of wild-type and *lys11* mutants.

We took our analysis a step further and investigated the functionality of the arbuscules in wild-type and mutant roots. For this, we performed two different assays, qRT-PCR investigation of AM symbiosis marker gene expression levels, and measurement of phosphorus levels in mutant and wild-type shoots using inductively coupled plasma-optical emission spectrometry (ICP-OES). Three different AM regulated genes were chosen for transcript level analyses. *PT4* expression level is generally used as an indicator for the phosphate import activity from the fungus into the plant^43^,^44^, while the transcript levels of *SbtM1* and *BCP* are general indicators of normal stage transition during arbuscule development^45^,^46^. These were supplemented by the analyses of *Lys11* and *RiGAPDH* expression levels, the last target being used as an indicator for the fungal mass in the same samples. All plant and fungal marker genes were found significantly increased in the inoculated samples, indicating a successful AM colonization (Fig. 4D). The transcript level of *Lys11* was significantly higher in wild-type roots compared to mutants, as it would be expected due to the effect of LORE1 retroelement insertion in *Lys11* gene. However, despite this difference in *Lys11* expression, we found no significant differences between *lys11* and wild-type samples regarding the amount of AM fungal mass, *PT4, BCP* or *SbMt1* transcript levels (Fig. 4D). These results confirm our observations from the independent microscopic quantification of AM colonization frequency in root fragments showing similar phenotypes for wild-type and *lys11* mutants. The analysis of phosphorus level in mutant and wild-type shoots revealed an increase in phosphorus content in the inoculated plants compared to the uninoculated. However, no significant differences were detected between mutant and wild-type samples regardless of AM inoculation (Supplementary Fig. S8C).

Taken together the results obtained from detailed quantitative and qualitative analyses of *lys11* mutants, we conclude that AM fungi colonize these mutants effectively, and that no detectable functional difference compared to wild-type was observed under the growth conditions used.

### Concomitant mutation of *Nfr1, Nfr5* and *Lys11* does not affect the AM symbiosis

Our results obtained from detailed phenotypic analyses showed that *lys11* mutants have normal root symbioses with rhizobia and AM fungi, even though *Lys11* has an AM-induced transcription, and has the ability to perceive LCOs. A possible explanation for the lack of defective AM symbiosis in *lys11* could be redundancy with NFR5, and possibly with NFR1 which previously was shown to contribute to AM infection in *L. japonicus*^10^,^31^. Therefore we investigated the expression patterns of *Nfr1* and *Nfr5* during AM symbiosis by relative quantification of transcripts, and analysis of promoter activities in roots using the same transcriptional reporters that were previously characterised in nodulation studies (Kawaharada *et al*., submitted). We found that *Nfr1* had a very low transcript level and a promoter activity that was below visual detection in mycorrhizal roots. *Nfr5*, on the other hand, had a higher level of expression that remained constant during AM infection (Supplementary Fig. S9). To further test the possibility of functional redundancy between Nod factor receptors and *Lys11* we obtained a triple mutant *nfr1*-*1nfr5*-*2lys11*-*3 (nfr1nfr5lys11*) and analysed its ability to sustain AM symbiosis. Similar to *lys11* single mutants, three different inoculum types and densities have been used for phenotyping the triple mutant. Quantitative analysis of the degree of mycorrhization and arbuscule abundance revealed that in these conditions, there was no significant difference between *nfr1nfr5lys11* plants and the corresponding controls (Methods) (Fig. 4E, Supplementary Fig. S8D,E). Further analysis of arbuscule morphology did not reveal obvious differences among genotypes (Fig. 4F,G).

These results obtained from the triple mutant analyses indicate that AM symbiosis in legumes is not directly controlled by NFR1, NFR5 and LYS11, and possibly additional component(s) contribute to secure a successful AM colonization in legume roots in their absence.

### LYS11 and NFR5 have different sensitivities to LCO decoration

Arbuscular mycorrhizal fungi produce a diverse set of chitin-derived molecules that vary in size and decorations, but their individual role in AM fungi-host signalling is unknown. We found that LYS11 can perceive LCOs, and is induced in specific root cells competent for AM symbiotic interface formation, pointing towards perception of such a ligand at that particular stage. This prompted us to further characterize LYS11 specificities for LCO structure using the root nodulation as a stable and robust assay. For this we analysed the efficiency of *Lys11* restoration of nodulation in *nfr5*-2 after rhizobial inoculation with rhizobial strains synthesizing Nod factor lacking decorations at the reducing end. We used the *M. loti nodZ* mutant producing Nod factors without the acetylated fucosyl, and *R. leguminosarum nodDABCIJL*^47^ producing Nod factors (LCO-V (Ac, C18:1)) similar to an AM-isolated LCO (LCO-V (C18:1)) (Supplementary Fig. S10A)^10^. We found that expression of *Nfr5* restored the *nfr5*-*2* defective nodulation, but only in the presence of *M. loti nodZ* strain indicating that *R. leguminosarum* minimal LCOs are either not perceived by NFR5 or that they are not able to induce nodule organogenesis. On the other hand, 35S- or *Nfr5*-driven *Lys11* could not restore nodulation of the *nfr5*-*2* plants when inoculated with any of these rhizobial strains (Supplementary Fig. S10B). These results show that LYS11, and NFR5 have different requirements for the LCO decorations, and that for LYS11 the presence of the acetylated fucosyl on the reducing end of LCO is required in order to allow an efficient downstream signalling in root nodule symbiosis.

## Discussion

The isolation of Myc factors and the finding that their acylated versions have structures analogues to Nod factors launched the hypothesis that Myc-LCO receptors would be similar in structure and function to Nod factor receptors, NFR1/LYK3 and NFR5/NFP, known from the model legumes *Lotus* and *Medicago*, respectively. Phylogenetic studies revealed that *Nfr5*/*Nfp* have each a paralogue, *Lys11*/*Lyr1*. We have investigated the function of *Lys11* in order to understand its role during root symbioses. The *Nfr5*/*Nfp* and *Lys11*/*Lyr1* are the result of segmental genome duplication in legumes. We found that despite their divergent evolution, LYS11 like NFR5 is a plasma membrane localized receptor that can interact with NFR1 in *N. benthamiana* leaves, perceive LCOs produced by *M. loti*, and induce nodule formation in the *nfr5*-*2* mutant when constitutively expressed. Differences in their extra- and intra-cellular domains resulted in a more robust Nod factor perception and intracellular signalling for the NFR5 compared to LYS11. This differed from the *Nfr1* close-related receptors, *Lys2, Lys6*, and *Lys7*, where none were able to complement the corresponding defective mutant phenotype when constitutively expressed^48^. Regardless of LYS11 innate capacity to perceive Nod factors, its expression, unlike *Nfr5*, was not detected in uninoculated (Supplementary Fig. S4, ref. 37), or *M. loti* infected roots, and did not contribute to nitrogen-fixing symbiosis, as shown by our phenotypic analyses of the mutants. These findings explain the strong null mutant phenotype resulting from mutation of *Nfr5* during nitrogen-fixing symbiosis^28^,^49^. Based on the similar strong phenotypes observed for *nfp* and *sym10* mutations in *Medicago* and pea^28^,^50^ we infer that their corresponding *Lys11* orthologues have undergone similar evolutionary changes as those we identified in *Lotus*.

Previous studies of receptor gene families revealed that promoter regions are prime sites for evolutionary divergence^51^. Our study shows that LYS11 and NFR5 have the capacity to perform a similar molecular function in cells where they are expressed, but it is their expression pattern that was mostly affected during evolution (Kawaharada *et al*., submitted). We found that *Lys11*, unlike *Nfr5* was specifically induced in the inner cortical cells either with arbuscules or in their close vicinity. This provided us with the location of LYS11 signalling and a plausible link to its function, induction of arbuscule formation. Such a function in *Lotus* would have been in line with the results obtained in *Parasponia* and tomato, which, according to Op den Camp *et al*., 2011 and Buendia *et al*., 2015 have only one *Nfr5*/*Lys11* gene-type, and when down-regulated by RNAi results in impairment of arbuscule formation or fungal infection, respectively^33^,^34^. Our results based on different types and concentrations of AM inoculum, and plant growth systems however did not point towards such a direct function of *Lys11* in presymbiotic signalling or arbuscule formation in *Lotus*. We show that this is not a result of redundancy between Nod factor receptors and *Lys11*, based on the observation that a triple mutant *nfr1nf5lys11* maintains the ability to efficiently form symbiosis with arbuscular mycorrhizal fungi.

These results from *Lotus, Medicago, Parasponia*, rice and tomato have nevertheless shown that impairment of LysM receptor kinases does not eradicate the presymbiotic signalling initiating arbuscular mycorrhiza invasion of roots^31^,^33^,^34^. Therefore different but not mutually exclusive scenarios could be envisaged for the role of *Lys11* in *Lotus*-AM symbiosis. The expression of *Lys11* is induced during intraradical AM colonization, in a manner similar to other AM-inducible genes. Conceptually, this opens a possible role for LYS11 in a two-step recognition mechanism reminiscent of the EPR3 perception of exopolysaccharides during rhizobial nodulation^52^. Following this line of thought, recognition of presymbiotic chitin-type signal(s) by an unknown receptor would control mycorrhizal infection and subsequently LYS11 would perceive similar signal(s) produced by the accommodating fungal hyphae. In the case of AM induced genes analysed so far, like for example phosphate transporters, mutation has a phenotypic impact on arbuscule morphology and/or function^44^,^53^. This was not observed for *Lys11* mutants, and based on the observation that its expression is induced behind the growth front of the fungal hyphae, rather than ahead of the infection, as it was observed for *Epr3* during rhizobial infection (Kawaharada *et al*., submitted), we suggest that an active AM symbiosis might be required for *Lys11* induction. In this scenario, LYS11 could fine-tune the efficiency of AM symbiosis. Our standard gnotobiotic growth conditions coupled with the obligate nature, profound, asynchronous and recurrent infection by the AM fungus would most likely conceal such subtle function of *Lys11*. Arbuscular mycorrhizal fungi undergo a massive and continuous morphological reorganization of their chitin-based cell wall during different stages of host infection^54^. Moreover, the arbuscules are short-lived structures whose degeneration, at least theoretically, exposes the host cell to a large amount of fungal wall-derived molecules. This continuous reorganization is therefore likely to result in a diverse panel of chitin-type signals^54^. In an alternative scenario, these various forms of chitin signals could be part of a complex strategy where collective and/or complementing activities ensure a robust and unselective AM symbiosis of 80% of land plants. Our results from *Lotus* showing an unperturbed capacity for AM infection and symbiosis in legume plants with three LCO receptors impaired concomitantly are in line with such strategy.

## Methods

### Plant material

Wild-type plants of *Lotus japonicus* ecotypes Gifu, MG20, or from a cross of these two ecotypes (Gifu x MG20) were used as controls. Homozygous *lys11* mutants were bred from heterozygous LORE1 insertion lines (ecotype Gifu) or TILLING lines (ecotype MG20). Homozygous triple mutant plants *nfr1nfr5lys11* were bred from a cross between *nfr1*-*1nfr5*-*2* double mutant (ecotype Gifu) and *lys11*-*3* (ecotype MG20). Composite plants^55^ were transferred to the appropriate analysis system after hairy roots emergence. Infiltration of *Nicotiana benthamiana* leaves using bacterial cultures of the transformed *Agrobacterium tumefaciens* was performed as previously described^39^. Infiltrated leaves were examined after 2–3 days using a Zeiss LSM510 MetaConfocal microscope.

### Rhizobial inoculation

#### Nodulation and infection thread assay

Plants were grown on 1/4 B&D media at 21 °C (light condition: 16 h/8 h day/night), and infection threads were counted per centimetre of root, after 9 and 14 days. 10 plants were analysed for each genotype and timepoint. For the nodulation assay plants (wild-type n = 18, *lys11*-*1* n = 28, *lys11*-*2* n = 22) were grown in granulated clay (leca), in the greenhouse and inoculated with wild-type *M. loti*. Nodules were counted after 3 weeks.

#### Complementation and expression analysis

Composite plants (wild-type/mutant shoots with transformed hairy roots) were transferred to magentas containing a 4:1 mixture of leca:vermiculite, 80 mL 1/4 B&D media and inoculated with rhizobia (OD_600_ = 0.01–0.02). *R. leguminosarum nodABCDIJL*^47^ was also used for LCO specificity analysis. Addition of the *NodL* to the *R. leguminosarum nodABCDIJ* improves the amount of LCO produced by this strain^47^ and therefore reduces the risk of negative results due to the low amount of LCOs. Roots were harvested 5 weeks after inoculation for complementation studies, whereas roots were harvested at 0, 7, 14 and 21 days after inoculation for expression studies. A detailed overview of the different strains used for the various experiments is presented in Supplementary Table S2.

### Mycorrhizal inoculation

#### Fungal material and mycorrhizal setups

Mycorrhizal colonization was monitored using three different systems and four different inocula. A detailed overview of the different systems used for the various experiments is presented in Supplementary Table S3.

**System (I**) *Rhizophagus irregularis* (DAOM 197198) was propagated in a chive nurse pot system (Supplementary Table S3). Chives were grown in a 3:1:1 v/v mixture of vermiculite, quarts (0–0.4 mm) and soil. Non-mycorrhized chives was similarly grown and used for mock-inoculation. Nurse pots with a maximum age of 10 weeks were used.

**System (II**) Sandwiches are composed of two nitrocellulose disks (MontaMil^®^ Membrane Filters (Frisenette: Cat. No. MCE047022), pore diameter 0.22 μm) enclosing both seedlings and AM spores^56^. Membranes were planted in sterile quarts (0–0.4 mm) in magenta. Mycorrhization of wild-type plants was monitored weekly to ensure that the analysis of the whole batch was performed when a colonization rate of 40–60% was achieved in wild-type roots.

To study the presymbiotic stages, composite plants were grown in single sandwiches (SS) and double sandwiches (DBS) as described in ref. 41. In the DBS composite plants were placed between two nitrocellulose membranes, and spores were placed at both external sides of the sandwich, covered by another membrane. SS without spores were used for mock-inoculation. The mycorrhizal branching on both SS and DBS was examined with a stereomicroscope to ensure a sufficient degree of mycorrhizal activity in the systems.

**System (III**) Spores were propagated in a split two-pot system, adapted from^57^: Both upper and lower pot contained a 1:1 mixture of vermiculite and sand (0–0.4 mm); additionally the lower pot was topped with a thin layer of sand and 5 mL spore inoculum (3500 spores). The upper pot contained ten wild-type (Gifu) plants and the bottom was covered with a nylon mesh (41 uM, Filtrade Aps., www.filtrade.dk) to prevent direct plant-fungal contact. Spores were primed for germination by maintaining them in contact with plant exudates for 3 weeks. The lower pot containing germinated spores was used for inoculation of six 1-week old plants grown in a separate new upper pot (no nylon mesh) that had to be analysed for AM symbiosis phenotype. Roots were exposed to the primed spores for 3 weeks. 3 biological replicates were performed using spore inoculum from different production lot batches.

### Quantification of mycorrhizal colonization

The estimation of mycorrhizal parameters was performed as described previously^58^. Five parameters were considered: (1) F%, percentage of segments showing internal colonization (frequency of mycorrhization); (2) M%, average percentage of colonization of root segments (intensity of mycorrhization); (3) m%, average percentage of colonization within infected areas; (4) a%, percentage of arbuscules within infected areas; (5) A%, percentage of arbuscules in the whole root system. Whole root systems were scored when using System II. 75 random root pieces (1 cm) were scored per replicate (225 cm/genotype) when using system III.

### Elicitor treatment

Composite plants (Gifu; *pLys11*:GUS) were grown in 1/4 B&D media for 21 days before subjected to monosolutions of different chitin oligomers: CO2, CO3, CO4 and CO5 (10^−6^ M), and *M. loti* NF (10^−8^ M). Plants (n = 6 for each treatment) were incubated for 24 h in the different monosolutions at room temperature before the histochemical analysis.

### Histochemical analysis of root tissue

Root samples from *Lotus japonicus* composite plants transformed with the GUS-reporter gene were covered with freshly prepared buffer [0.1 M sodium phosphate buffer (pH 7) 0.5 mM K_4_Fe (CN)_6_, 5 mM K_3_Fe(CN)_6_, 0.3% Triton X, 0.3% X–Gluc] and incubated at 37 °C in the dark. To visualize AM fungal structures, either whole root or GUS-positive segments alone were cleared in 10% KOH for 10 min, and counterstained with 1 μg/ml WGA Alexa Fluor 488 (Molecular Probes) overnight in vacuum. Samples were observed under an optical fluorescent microscope (Zeiss Fluorescence). For thin-sectioning, tissue samples were embedded in Technovite 7100 resin (Hereus Kulzer, Wehrheim, Germany) following the manufacturer’s instructions. 5–6 μm-thick sections were prepared using a LEICA RM2045 microtome. Sections were stained with 0.2% ruthenium red to visualize plant cell membranes.

### Shoot phosphorous measurements

10–20 mg dried leaf material from non-mycorrhized and mycorrhized *Lotus japonicus* wild-type and *lys11* mutant plants were digested in 1 mL 6 M HNO_3_ at 90 °C with shaking (450 rpm) for 1 hour. The leaf extract was diluted to 6 ml with distilled water and filtered. All experiments were performed in three replicates and blanks (containing no samples) were simultaneously run. Phosphorous concentration was determined with a Perkin Elmer Optima 7000 (Perkin Elmer, Norwalk, Connecticut, USA) inductively coupled plasma-optical emission spectrometer (ICP-OES). The reagents adopted were of analytical grade purity. Metal standard solutions were prepared from concentrated stock solutions (Merck Titrisol). High purity water (HPW) obtained from a Milli-Q apparatus (Millipore, Bedford, USA) was used throughout.

### RNA isolation, cDNA synthesis and real-time RT-PCR

Roots and shoots of mycorrhiza- and mock-inoculated plants were harvested and immediately transferred to liquid Nitrogen.

#### Transcript quantification of Nfr1, Nfr5 and Lys11 in wild-type or lys11 mutants

Relative quantification of transcripts amplified with specific primers (Supplementary Table S5) and normalized to a calibrator was performed as described previously^37^. All transcripts were normalized to three housekeeping genes (ATP synthase, ubiquitin-conjugating enzyme, and protein phosphatase 2A).

#### Transcript quantification of Castor and Pollux in the sandwich systems

Root samples from two biological replicas were collected at 3 dpi. RNA isolation, cDNA synthesis and qRT-PCR on ICyclerBioRad was performed as previously described^59^. Expression levels were normalized to *LjUbiquitin* level. A constitutively expressed gene from *G. margarita*, the elongation factor GI:37653264 (*Gm*EF) was included as a control for the separation of plant and fungus by the membrane in the double sandwich and for quantification of AM level of colonization.

### Plasmid constructs and cloning strategies

#### Gateway cloning

A putative *Lys11* promoter region of (1565 bp), and the coding sequences of *Lys11* (1776 bp) and *Lys15* (1809 bp) were amplified from *Lotus japonicus* Gifu genomic DNA and used for Gateway cloning in pIV10:GW:GUS:Nos, and pIV10:35S:GW:35S for hairy root transformations and in pGREEN029:35S:GW:nYFP/cYFP or pEarlyGate101:35S:GW:YFP for protein localisation and protein-protein interactions in *N. benthamiana*. The resultant constructs were transformed into either *A. rhizogenes* AR12 or AR1193, or *A. tumefaciens* (AGL1). *GoldenGate cloning*: Modules for *Nfr5* promoter, *Lys11* or *Nfr5* extracellular or intracellular regions, and *Nfr5* terminator were amplified and cloned individually. The final versions of the constructs N5-N5, L11-L11, N5-L11, L11-N5 were obtained by specifically combining the corresponding extracellular and intracellular modules as described previously (ref. 60, Supplementary Table S4). L11-L11 is identical to *Lys11* construct obtained by Gateway cloning, whereas N5-N5 differs from the Nfr5 construct at G717A, with no consequences for the aminoacid sequence. The resultant constructs were transformed into *A. rhizogenes* AR1193 or AR12^55^. All constructs were sequence-verified in *E. coli*. A detailed description of the constructs is presented in Supplementary Table S4.

## Additional Information

**How to cite this article**: Rasmussen, S. R. *et al*. Intraradical colonization by arbuscular mycorrhizal fungi triggers induction of a lipochitooligosaccharide receptor. *Sci. Rep.* **6**, 29733; doi: 10.1038/srep29733 (2016).

## Supplementary Material

Supplementary Information
