# Supplementary material for: Intraradical colonization by arbuscular mycorrhizal fungi triggers induction of a lipochitooligosaccharide receptor
Source: Sci Rep. 2016 Jul 20;6:29733. doi: 10.1038/srep29733 (PMC4951684; doi:10.1038/srep29733)
Supplement: Supplementary Information [file srep29733-s1.pdf]

Intraradical colonization by arbuscular mycorrhizal fungi triggers induction of a lipochitooligosaccharide receptor

Rasmussen S. R.[1 †], Füchtbauer W.[1†], Novero M.[2], Volpe V.[2], Malkov N.[1], Genre A.[2], Bonfante P.[2], Stougaard J[1]. and Radutoiu S.[1*]

1. Department of Molecular Biology and Genetics, Centre for Carbohydrate Recognition and Signalling, Aarhus University, Denmark
2. Department of Life Science and Systems Biology, University of Torino, Italy

† These authors contributed equally to this work

Corresponding author:
Simona Radutoiu
radutoiu@mbg.au.dk

Supplementary Information

Supplementary Figure S1: Alignment of NFR5 and LYS11 aminoacid sequences.

The predicted signal peptide is marked in red, the three LysM domains in yellow, the transmembrane region in green and the kinase in blue (domains are based on PROSITE and SMART databases and Madsen et al. 2003). Conservation indicates identical amino acids as peaks. L118 in NFR5 [1] is marked with * and S282 in NFR5 [2] with #.

Supplementary Figure S2: LYS11 performs similar functions as NFR5 when expressed in *N. benthamiana* heterologous system.

(A) Confocal image showing LYS11-eYFP and the plasma membrane marker PIP2A-mCherry co-

localizing at the plasma membrane [3] of *N. benthamiana* leaf cells. (**B**) Plasmolysis showed that LYS11 follows the plasma membrane (arrows indicate the location where plasma membrane has separated from the cell wall). YFP signal is reconstituted at the plasma membrane when co-expressing NFR1-T481A-cYFP and LYS11-nYFP (**C**) or NFR1-T481A-cYFP and NFR5-nYFP (**D**). (**E**) Co-expression of LYS11 with NFR1, but not with NFR5 initiates a downstream signalling leading to cell death (area marked by dashed line in **D**). Arrows indicate the infiltration spots. Scale bars represent 100 μ m (**A-D**) and 2cm (**E, F**).

Supplementary Figure S3: Lys11 complements the nodulation phenotype of *nfr5-2* when overexpressed. Composite *L. japonicus* plants with hairy roots obtained as described previously[1,4.] Nodule formation observed on the transformed root of *nfr5-2* overexpressing *Nfr5* (left) or *Lys11* (center). The right panel shows a representative plant from the three experiments where no complementation was observed on the *nfr5-2* plants; the empty vector, *Lys11* controlled by the promoter of *Nfr5* (*pNfr5*), or when *Lys15* was overexpressed (*p35S*:*Lys15*). Asterisks indicate the untransformed *nfr5-2* mutant root. The transformed roots are stained blue (right) as the AR12 *A. rhizogenes* strain used for transformation expresses the 35S:GUS. Scale bars represent 2cm.

Supplementary Figure S4: Lys11 gene expression is not regulated by rhizobia. (**A, B**) A basal low level of *Lys11* promoter activity (arrow) is occasionally observed in the vascular tissue of the main root, and in the root cells at the base of lateral root primordia of both mock- and *M. loti*-inoculated roots. (**C**) *Lys11* promoter activity is occasionally observed in the vascular tissue of the nodule in the *M. loti*-inoculated roots. *Nfr5* promoter is highly active in uninoculated roots (arrow) (**D**), inoculated roots (arrow) and young nodule primodia (arrowhead) (**E**), and has a lower activity in maturing nodules (arrowhead) (**F**). The *pNfr5*:GUS construct is described in Kawaharada *et al.,* submitted. Scale bars represent 100 μ m.

Supplementary Figure S5: *lys11* mutant plants have normal nodulation phenotype.

Normal plant growth (upper panels), and root nodule development (lower panels) observed on *lys11-1* and *lys11-2* mutant and wild-type plants. Scale bars represent 10mm (top) or 1mm (bottom).

Supplementary Figure S6: *Lys11* gene expression is regulated by AM fungus. *Lys11* promoter activity is observed in *R. irregularis*- inoculated (Myc) roots (system I) harvested after 7, 14 and 21 days post inoculation (dpi), whereas no activity was observed in Mock- or after 1dpi *R. irregularis* inoculated roots. Note the increased expression of *Lys11* with prolonged time of fungal infection revealed by the enlarged zones and increased intensity of the GUS stain. Scale bars represent 10mm.

Supplementary Figure S7: Activation of *Lys11* promoter requires *SymRK* and *CCaMK*. Concomitant visualization of *Lys11* promoter activity (blue colour after GUS staining) and *R. irregularis* (fluorescence after WGA Alexa 488 staining) in the same root segments (system I) shows that Nod factor receptor mutants *nfr1-1* (**A**), *nfr5-2* (**B**), *nfr1-1nfr5-2* (**C**) and transcriptional activator mutant *nsp2-3* (**D**) support a normal activity of *Lys11* promoter activity (black arrow), as well as AM colonization and arbuscule formation (white arrow). By contrast, *symrk-2* (**E**) and *ccamk-3* (**F**) do not show any detectable *Lys11* promoter activity and only extraradical hyphae (*). Scale bars represent 50 μm.

Supplementary Figure S8: Arbuscular mycorrhizal fungus colonizes *lys11* and *nfr1nfr5lys11* mutants efficiently. Normal arbuscules are formed in *lys11-2* (**A**) and *lys11-3* (**B**) infected by *R. irregularis* (system II). Similar arbuscule morphology was observed for WT, *lys11* and *nfr1nfr5lys11* mutants when inoculated with *R. irregularis* (system III) or after inoculation with *G. margarita*. (**C**) WT and *lys11* mutants inoculated with *R. irregularis* (system II) accumulate similar but slightly higher level of phosphorous in the shoot when compared to mock samples. Kruskal-Wallis statistical test ($P < 0.05$) revealed no significant differences between genotypes or mock- versus Myc-inoculated. Quantification of AM infection in roots inoculated with *R. irregularis*, system III (**D**), or *G. margarita* (system II) (**E**) show no significant differences between corresponding wild-type and mutants. . F%,

M%, m%, a% and A% are defined in Methods. Scale bars present 50 μm (**A, B**). Error bars represent the standard deviation (**C**), and 95% confidence interval (**D, E**).

Supplementary Figure S9: *Nfr1* and *Nfr5* expression during symbiosis with arbuscular mycorrhiza. Concomitant visualization of *Nfr1* or *Nfr5* promoter activities (blue colour after GUS staining) and *R. irregularis* (fluorescence after WGA Alexa 488 staining) in the same root segments (system I) containing different stages of the AM symbiosis. *Nfr1* promoter activity is visually undetectable in these conditions (**A, C, E, G**), whereas *Nfr5* promoter activity (**B, D, F, H**) is observed in the roots regardless of the absence (**B**) or presence of AM fungus (**D, F, H**). There is no change in the expression patterns of *Nfr1* and *Nfr5* (black arrows) during various stages of fungal infection including hyphopodium formation (*) (**C, D**), progression of the intraradical hyphae (**E, F**) or arbuscule formation (white arrow) (**G, H**). (**I**) Transcript quantification of *Nfr1* and *Nfr5* in wild-type roots shows reduction of their level in *R. irregularis*-inoculated (Myc) roots (system II) versus Mock-treated. Scale bars represent 100 μm. Error bars in (**I**) show the 95% confidence interval.

Supplementary Figure S10. LCO specificity of NFR5 and LYS11 receptor proteins.
(**A**) The likely structures of the Myc-factor analogue produced by *R. leguminosarum nodABCDIJL* [5] compared to the non-sulphated Myc-factor produced by *R. leguminosarum nodABCDIJ* [6]. Addition of the *NodL* (marked with a red box in **A**) to the *R. leguminosarum nodABCDIJ* improves the amount of LCO produced by this strain [5] and therefore reduces the risk of negative results due to the low amount of LCOs (**B**) Average number of nodules formed by the *nfr5-2* mutant expressing *Lys11* or *Nfr5* after inoculation with *M.loti*, *M.loti nodZ* mutant or *R. leguminosarum nodABCDIJL* strains. Constructs were expressed under the control of CaMV 35S (*p35S*) or *Nfr5* (*pNfr5*) promoters. Error bars show the 95% confidence interval.

Supplementary Table S1. Induction of *Lys11* expression in transformed roots treated with various elicitors, *M. loti* and AM fungi.

Supplementary Table S2 Rhizobial strains used in this study.

Supplementary Table S3 Arbuscular mycorrhizal isolates used in this study.

Supplementary Table S4 Constructs and Transformations.

Supplementary Table S5 Primers for real-time RT-PCR

Supplementary References

Supplementary Figure S1: Alignment of NFR5 and LYS11 aminoacid sequences.

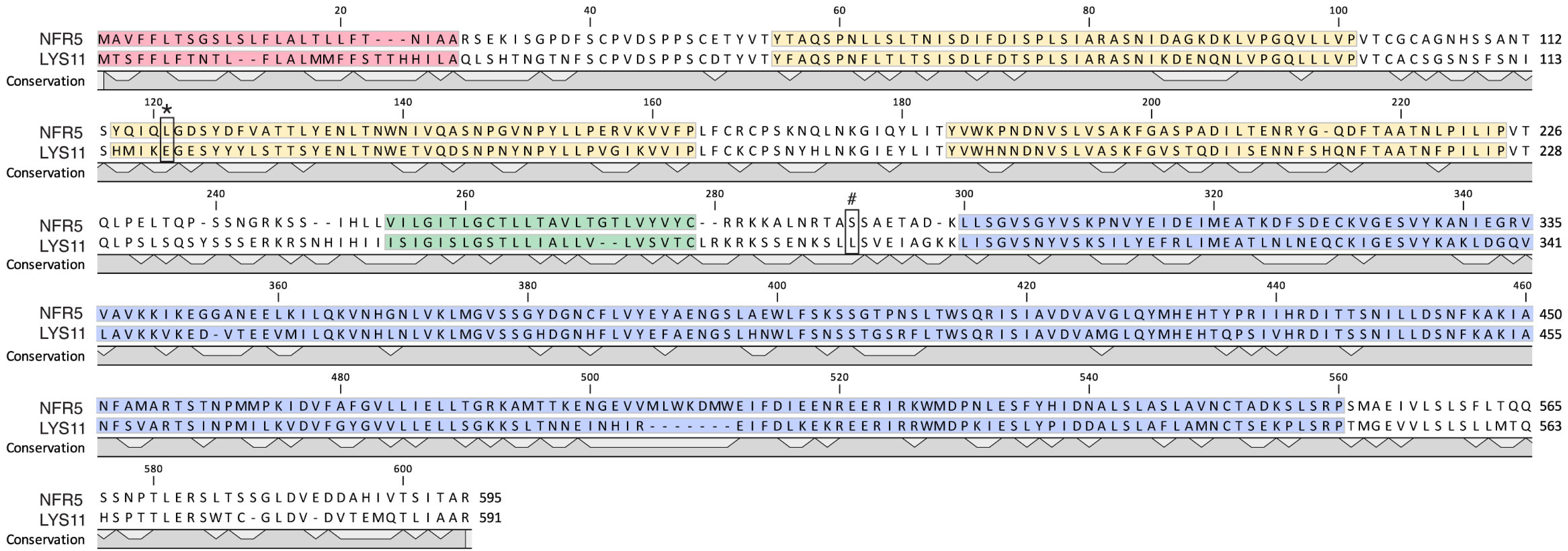

Supplementary Figure S2: LYS11 performs similar functions as NFR5 when expressed in *N. benthamiana* heterologous system.

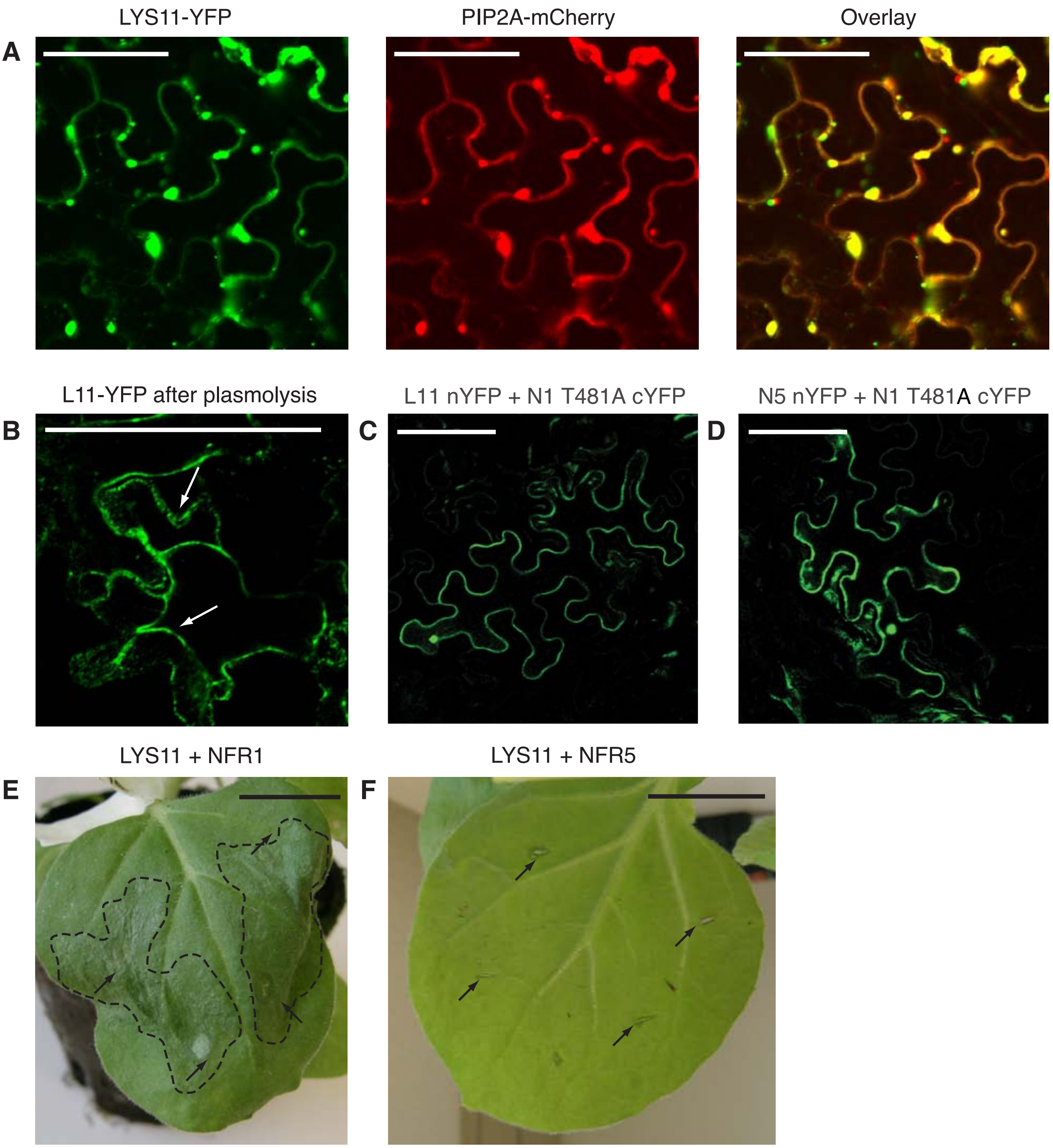

Supplementary Figure S3: *Lys11* complements the nodulation phenotype of *nfr5-2* when overexpressed. Composite *L. japonicus* plants with hairy roots obtained as described previously[1, 4].

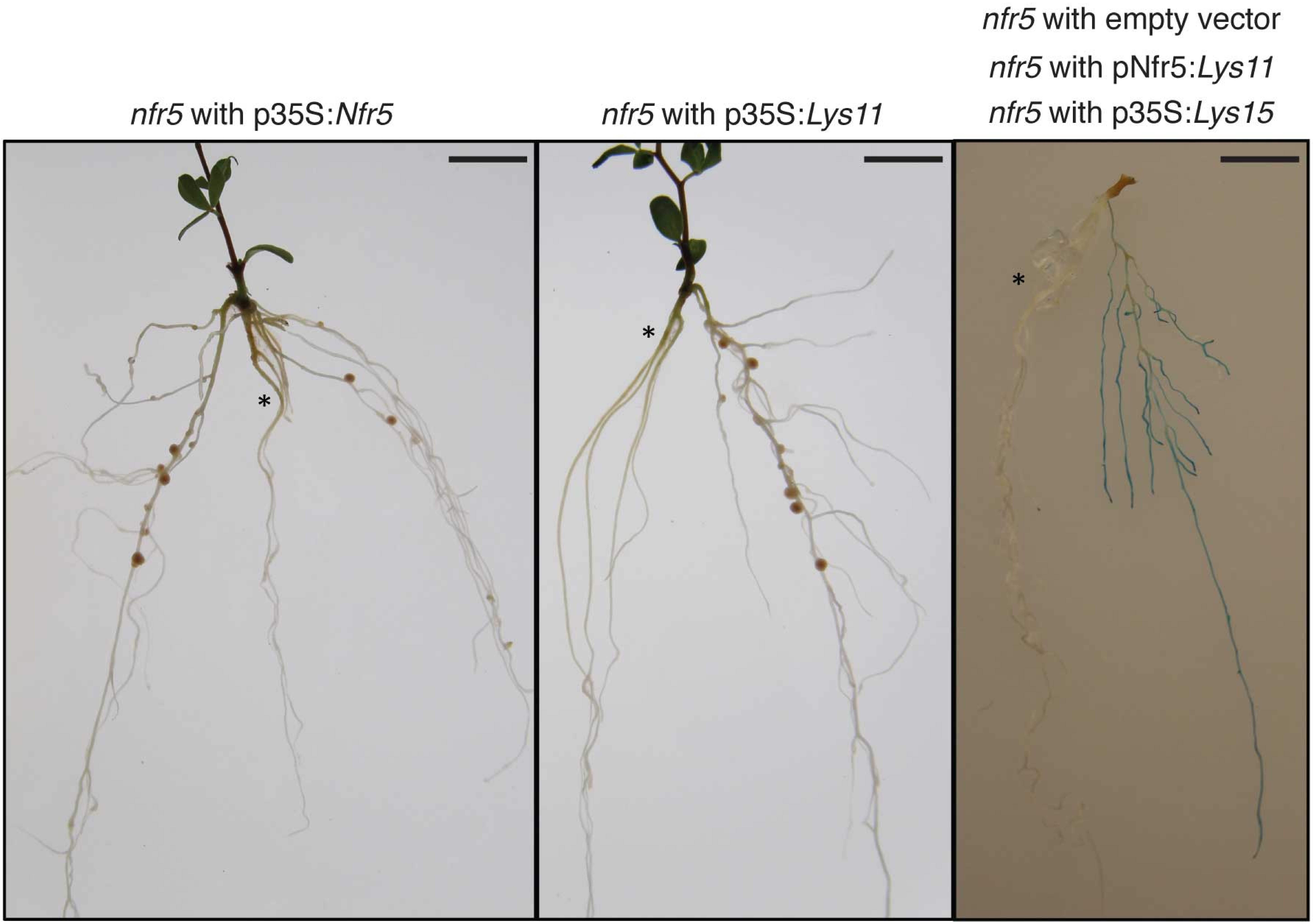

Supplementary Figure S4: *Lys11* gene expression is not regulated by rhizobia.

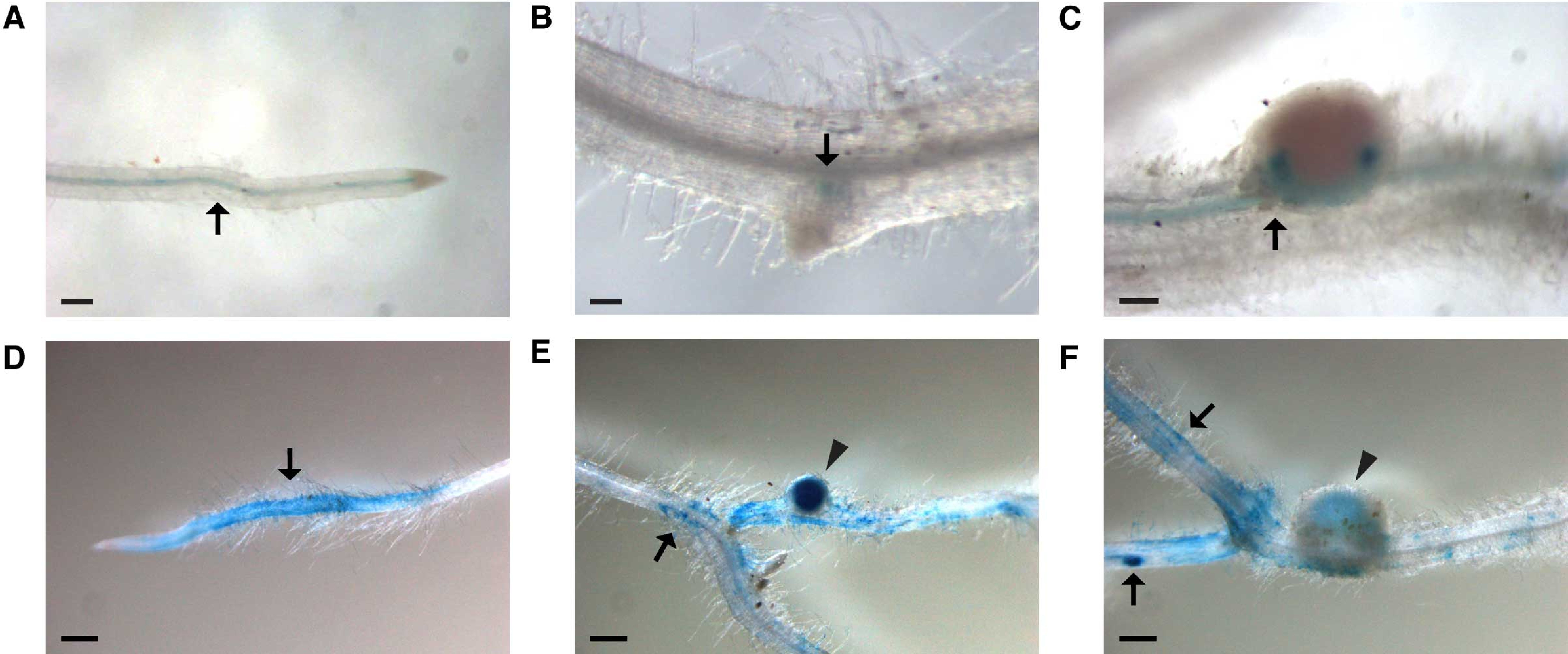

Supplementary Figure S5: *lys11* mutant plants have normal nodulation phenotype.

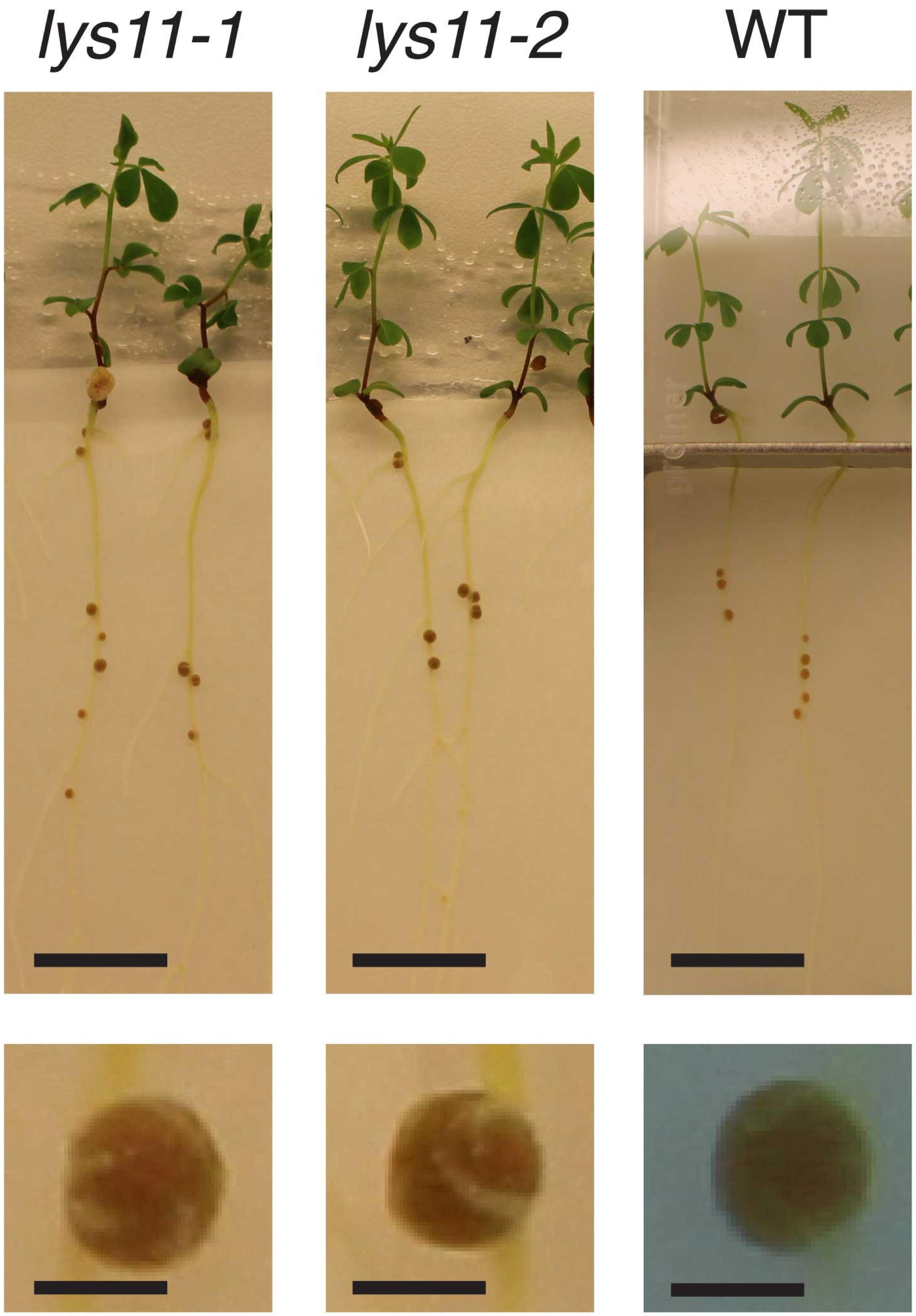

Supplementary Figure S6: *Lys11* gene expression is regulated by AM fungus.

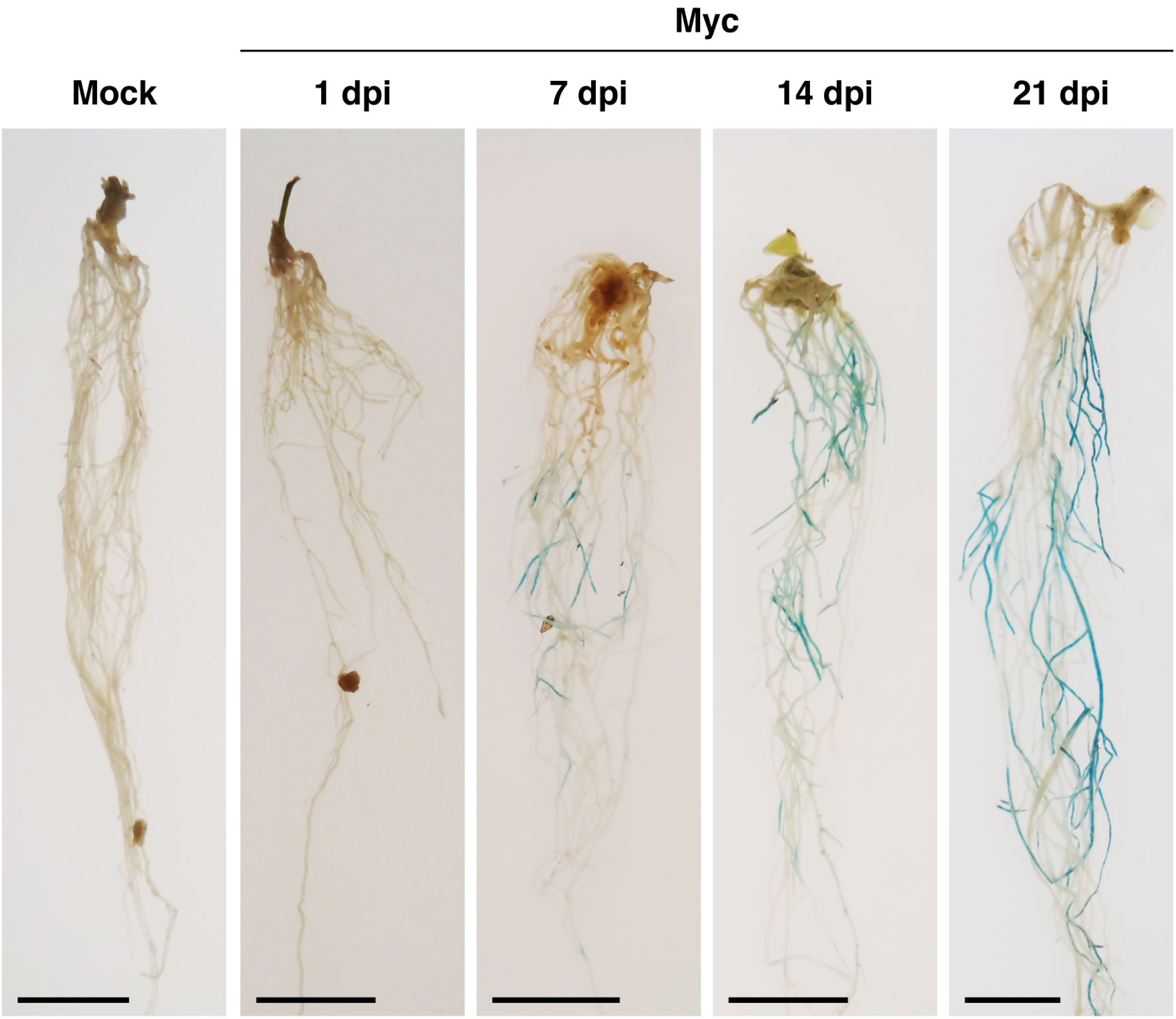

Supplementary Figure S7: Activation of *Lys11* promoter requires *SymRK* and *CCaMK*.

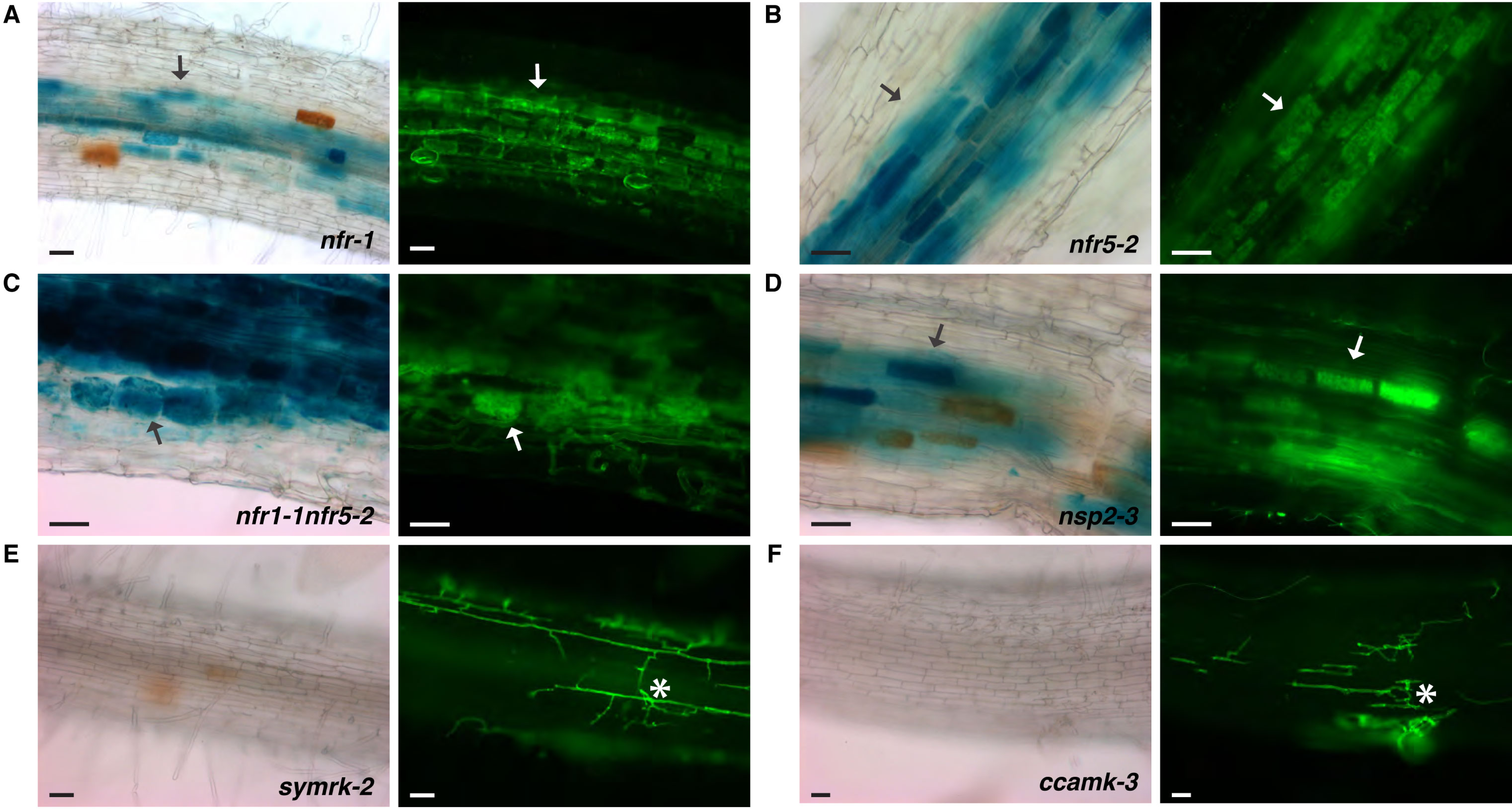

Supplementary Figure S8: Arbuscular mycorrhizal fungi colonize *lys11* and *nfr1nfr5lys11* mutants efficiently.

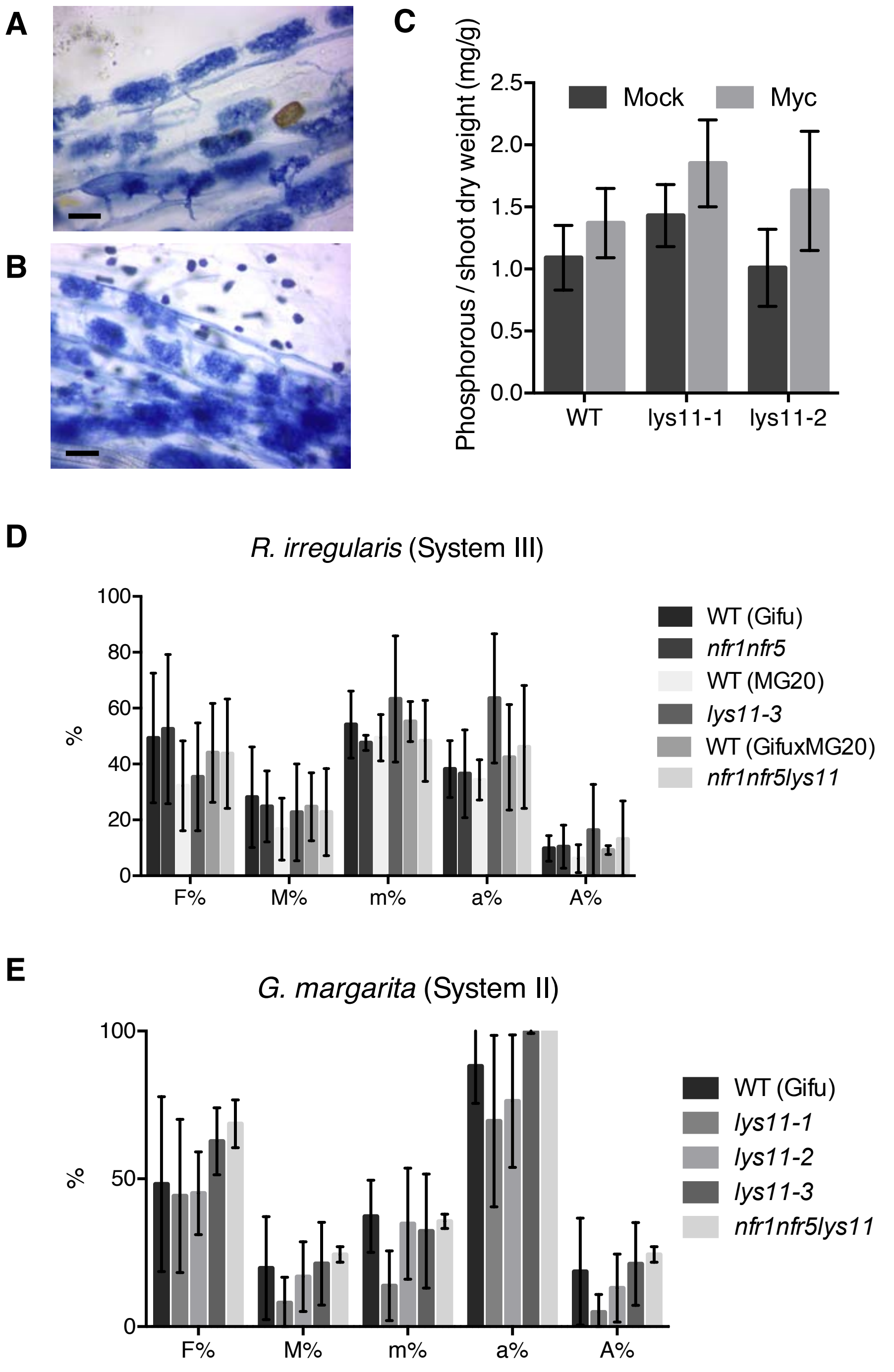

Supplementary Figure S9: *Nfr1* and *Nfr5* expression during symbiosis with arbuscular mycorrhizal fungi.

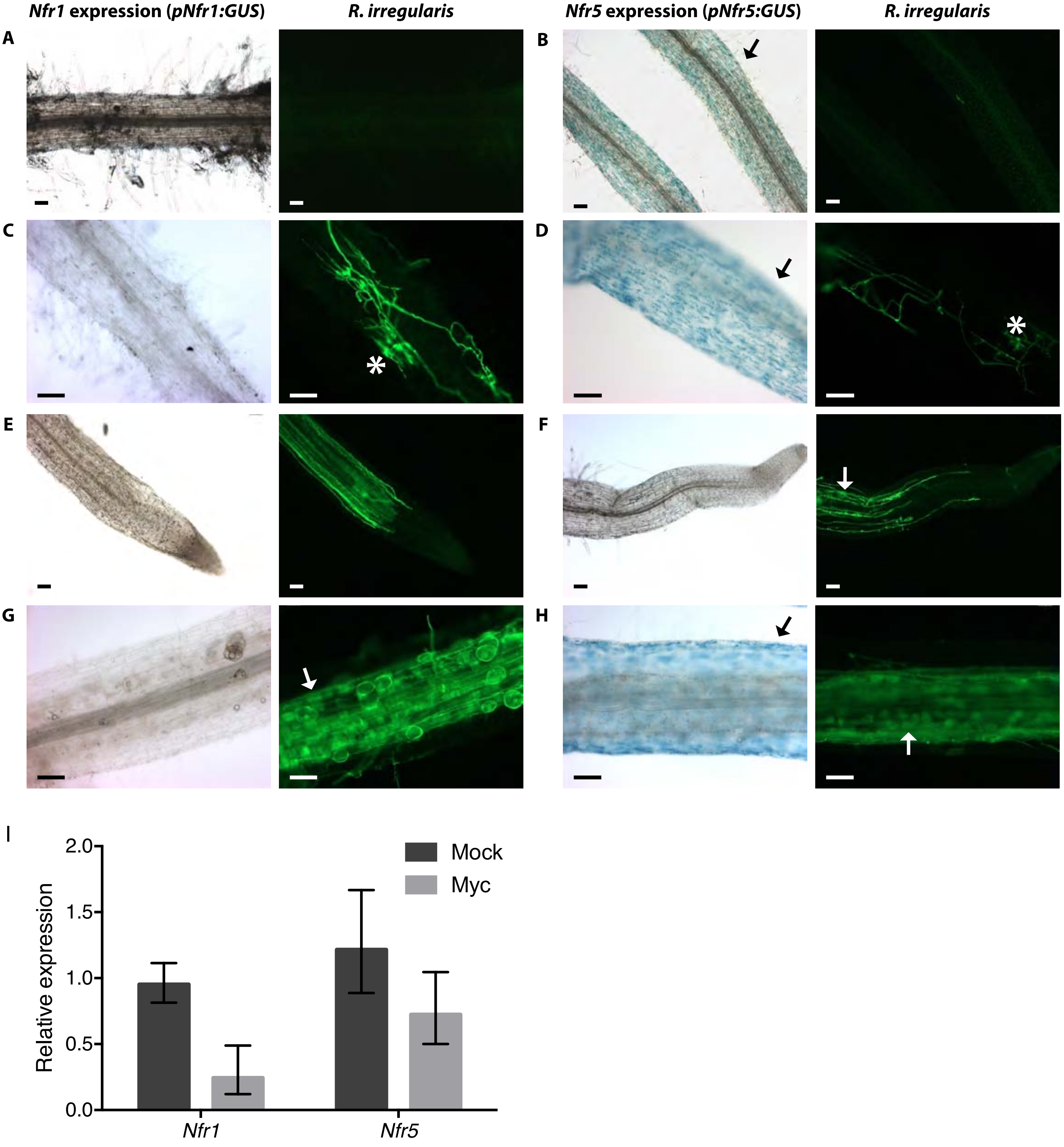

Supplementary Figure S10: LCO specificity of NFR5 and LYS11 receptor proteins.

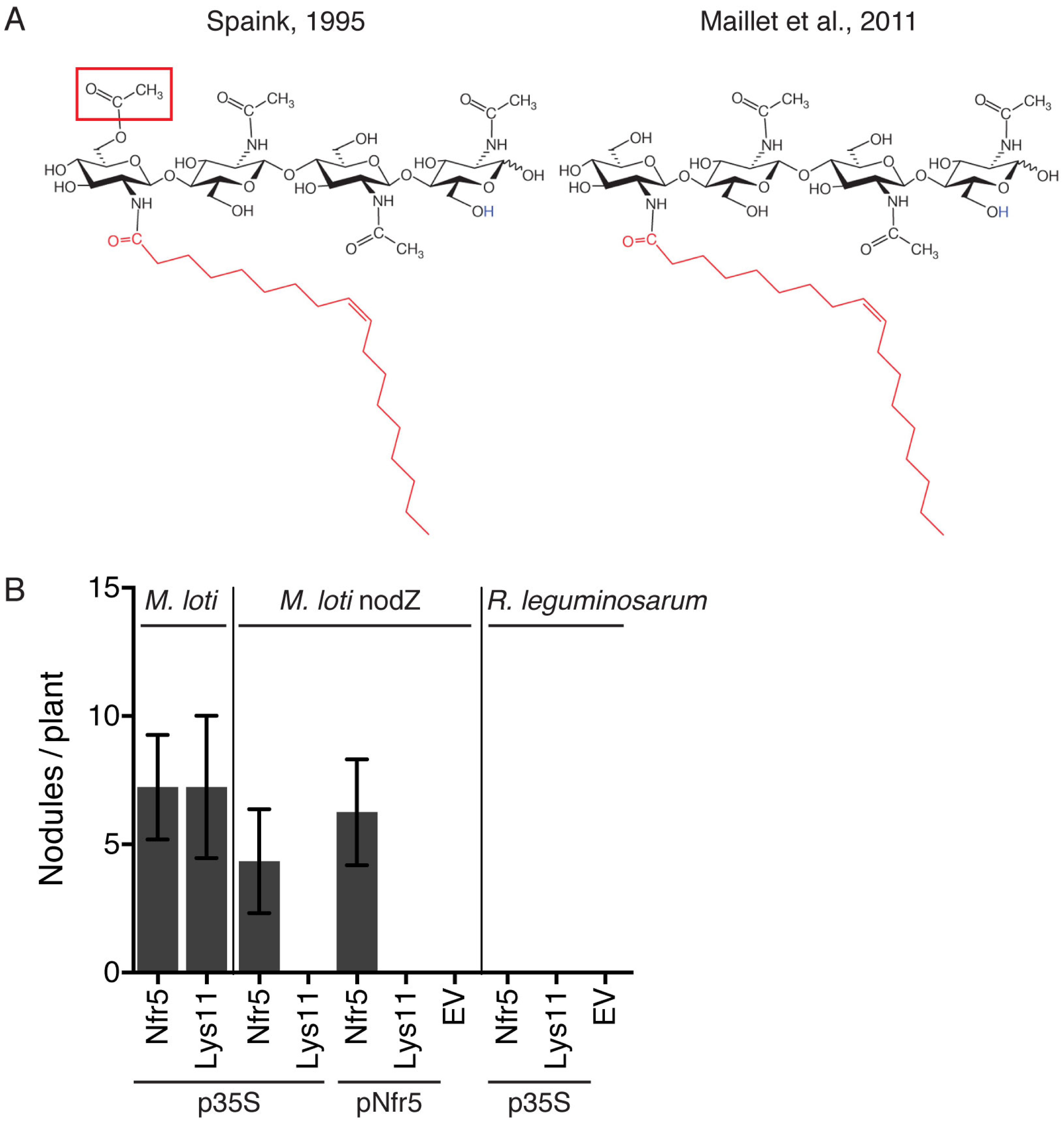

Supplementary Table S1: Induction of *Lys11* expression in transformed roots treated with various elicitors, *M. loti* and AM fungi.

**Treatment**	**Concentration**	**Number of plants tested (n)**	***Lys11* expression**	**Reference**
Mock		20	Undetected	A representative image of the roots is shown in Supplementary Figure S6 (Mock)
CO2	10^{-6}M	6	Undetected	
CO3	10^{-6}M	6	Undetected	
CO4	10^{-6}M	6	Undetected	
CO5	10^{-6}M	6	Undetected	
M. loti NF	10^{-8} M	6	Undetected	
M. loti	OD_{600}=0.015	20	Undetected	Supplementary Figure S4
G. margarita exudates (system II-DBS)	30 spores / 2 plants	8	Undetected	Fig. 2
G. margarita (System II-SS)	15 spores / 2 plants	8	Induced	Fig. 2
R. irregularis (system I)	Chive system	20	Induced	Supplementary Figure S6

Supplementary Table S2: Rhizobial strains used in this study

Organism	Strain	Experiment	Genotype	Reference
M. loti	MAFF303099	Promoter:GUS expression studies	Wild type Gifu	Kaneko et al., 2009 [7]
	MAFF dsRed	Infection thread quantification	Wild type Gifu, *lys11-1*, *lys11-2*	Markmann et al., 2008 [8]
	R7A	Complementation assay	*nfr5-2*	Sullivan and Ronson 1998 [9]
	R7A nodZ	Complementation assay	*nfr5-2*	Rodpothong et al., 2009 [10]
	NZP2235	Greenhouse nodulation assay	Wild type Gifu, *lys11-1*, *lys11-2*	Jarvis et al., 1982 [11]
R. leguminosarum	nodDABCIJL	Complementation assay	*nfr5-2*	Spaink et al., 1995 [5]

Supplementary Table S3: Arbuscular mycorrhizal isolates used in this study

Organism	Fungal inoculum	System	Experiment	Genotypes	Inoculation density	Time point (dpi)	n
R. irregularis	DAOM 197198 (1)	System I	***Lys11* expression analysis**				
		(chive)	Transcript quantification (RT-qPCR)	Wild type Gifu		2, 7, 14, 21	15
			Promoter-*GUS* expression	Wild type Gifu		1,7, 14, 21	20
				nfr1-1		28	20
				nfr5-5		28	20
				nfr1-1nfr5-2		28	20
				symrk-2		28	20
				ccamk-3		28	20
				nsp2-3		28	20
			***Nfr1* and *Nfr5* expression**				
			Promoter-*GUS* expression	Wild type Gifu (*pNfr1:GUS*)		1, 3, 7, 10, 14	8
			Promoter-*GUS* expression	Wild type Gifu (*pNfr5:GUS*)		1, 3, 7, 10, 14	8
R. Irregularis	BEG140 (2)	System II	**AM phenotyping**				
Symbiom Ltd, http://www.symbiom.cz/		(sandwich)	AM quantification	Wild type Gifu	30-40 spores/plant	32	9
				lys11-1	30-40 spores/plant	32	9
				lys11-2	30-40 spores/plant	32	9
				lys11-3	30-40 spores/plant	32	9
				Wildtype GifuxMG20	30-40 spores/plant	36	6
				nfr1-1nfr5-2lys11-3	30-40 spores/plant	36	6
			Transcript quantifcation (RT-qPCR) on	Wild type Gifu	50 spores/plant	28	9
			root and phosphorous measurement on	*lys11-1*	50 spores/plant	28	9
			shoot	*lys11-2*	50 spores/plant	28	9
			***Nfr1* and *Nfr5* expression**				
			Transcript quantification (RT-qPCR)	Wild type Gifu	50 spores/plant	28	9
G. margarita	BEG34 (3)	System II	**AM phenotyping**				
MycAgro Lab, http://mycagrolab.com/		(sandwich)	AM quantification	Wild type Gifu	20 spores/plant	28	9
				lys11-1	20 spores/plant	28	9
				lys11-2	20 spores/plant	28	9
				lys11-3	20 spores/plant	28	3
				nfr1-1nfr5-2lys11-3	20 spores/plant	28	3
G. margarita	BEG34 (3)	System II	***Lys11* expression analysis**				
(in house production)		SS	Promoter-*GUS* expression (4)	Wild type Gifu	15 spores/2 plants	7, 28	8
		DBS		Wild type Gifu	30 spores/2 plants	7, 28	8
		SS	Transcript quantification (RT-qPCR)	Wild type Gifu (*pLys11:GUS*)	15 spores/2 plants	3	4
		DBS		Wild type Gifu (*pLys11:GUS*)	30 spores/2 plants	3	4
R. Irregularis	009 (5)	System III	**AM phenotyping**				
Mycovitro SL, Glomygel ® Hortalizas, http://www.mycovitro.com/		(split-pot)	AM quantification	Wild type Gifu	3500 spores/6 plants	21	54
				Wild type MG20	3500 spores/6 plants	21	54
				Wild type GifuxMG20	3500 spores/6 plants	21	54
				lys11-3	3500 spores/6 plants	21	54
				nfr1-1nfr5-2lys11-3	3500 spores/6 plants	21	54

(1) *Rhizophagus irregularis* (DAOM 197198) [12] was propagated in a chive nurse pot system adapted from The Lotus Handbook[13].

(2) Single fungus-inoculum provided as a powder of spores mixed in diatomite. The powder was suspended in modified Long Ashton solution [14] and aliquoted onto the filter disks corresponding to the appropriate amount of spores. A corresponding suspension of diatomite was used for mock-inoculation.

(3) *Gigaspora margarita* isolate BEG 34 (International Bank for the Glomeromycota, University of Kent, UK)

(4) To ensure a nicely developed root system that could support AMF infection, composite plants were grown on M-medium (no sugar) [15] with 300 µg/mL cefotaxime for 14 days prior to inoculation.

(5) Single fungus-inoculum provided as a suspension of AM fungal propagules (spores, active extraradical hyphal pieces as well as mycorrhizal root pieces) in a mean total concentration of 6000 propagules per ml. *R. Irregularis* ecotype 009 (MYCOVITRO S.L., Granada, Spain) is *in vitro*-produced in a gel-patented substrate (patent hold by Consejo Superior de Investigaciones Cientificas, CSIC, Spain).

Plants grown in system I and III were watered weekly with Chive nurse pot nutrient solution [13] supplemented with 5 mM KNO3, while plants grown in system II were fertilized with modified Long-Ashton nutrient solution [14] containing 3.2 µM Na2HPO4. All plants were grown at 21◦C (light condition: 16 h/8 h day/night).

Mycorrhized roots were stained with either 5% ink in 5% acetic acid solution [16] or 0.1% cotton blue [17]

Supplementary Table S4: Constructs and Transformations

**Golden Gate cloning**			
**Constructs**	**Promoter(p)**	**Gene**	**Terminator(t)**
pNfr5:Lys11:tNfr5	1316 bp	1539 bp	432 bp
pNfr5:Nfr5:tNfr5	1316 bp	1785 bp	432 bp
pNfr5:Lys11:Nfr5:tNfr5	1316 bp	752 bp(*Lys11*):1069 bp(*Nfr5*)	432 bp
pNfr5:Nfr5:Lys11:tNfr5	1316 bp	716 bp(*Nfr5*):1051 bp(*Lys11*)	432 bp

**Organism**	**Strain**	**Experiment**	**Genotype**
A. tumefaciens	AGL1	*N. benthamiana* expression	
A. rhizogenes	AR1193	Promoter:*GUS*	Wild type Gifu
	AR12	Complementation	*nfr5-2*

Supplementary Table S5: Primers for real-time RT-PCR

Gene	Forward primer (5'-3')	Reverse primer (5'-3')	Reference
LjLys11	CTTAGCCTCTCCCTTCTCATGAC	CCGACTCTGACACGGACACTG	Lohmann et al. 2010 [18]
LjNfr1	GGCCCTTTCAACACAAGATG	TGTAGCCTTCGCTAGTTCCTG	Lohmann et al. 2010 [18]
LjNfr5	GGCCAGAACTTCGACCAAC	TCCTTCCACAGCATAACCAC	Lohmann et al. 2010 [18]
LjPT4	CCAGAACCTCACACAGAAAGACATC	AACACGGTGAACCAGTACCCTGG	This study
LjUBI	AACATTCAGAAAGAGTCCAC	TTACAAGCCACAACAATCAC	Volpe et al. 2013 [19]
LjCastor	TGATGGTGGCCTTGACATAA	TCGAGAAGTTTCCTCCCTGA	Imaizumi-Anraku et al. 2005 [20]
LjPollux	ACACCATAACCACCGCTCTC	GCAGAAAAGGCAAATGAGC	Imaizumi-Anraku et al. 2005 [20]
GmEF	TGAACCTCCAACCAGACCAACTG	GGTAAGACCAACTGGGGCGAATG	Salvioli et al. 2008 [21]
LjPP2A	GTAAATGCGTCTAAAGATAGGGTCC	ACTAGACTGTAGTGCTTGAGAGGC	Lohmann et al. 2010 [18]
LjUBC	ATGTGCATTTTAAGACAGGG	GAACGTAGAAGATTGCCTGAA	Lohmann et al. 2010 [18]
LjATP	CAATGTCGCCAAGGCCCATGGTG	AACACCACTCTCGATCATTTCTCTG	Lohmann et al. 2010 [18]
LjBCP	GTGTGGGTGACAACCTTGGGTTC	ATTTCCTCCCTTCAGTTGTTAATGG	This study
LjSbtM1	CAGGTGAACCAGAAGGTTGCATAC	AGCAGCACCCTCTCTATCTTCATGC	This study
RiGADPH	GACGTCTCAGTTGTTGATTTA	TTTGGCATCAAAAATACTAGA	Buendia et al. 2015 [19]

Supplementary References

1. Radutoiu, S. *et al.* LysM domains mediate lipochitin-oligosaccharide recognition and *Nfr* genes extend the symbiotic host range. *EMBO J.* **26,** 3923–3935 (2007).
2. Madsen, E. B. *et al.* Autophosphorylation is essential for the *in vivo* function of the *Lotus japonicus* Nod factor receptor 1 and receptor-mediated signalling in cooperation with Nod factor receptor 5. *Plant J.* **65,** 404–417 (2011).
3. Nelson, B. K., Cai, X. & Nebenführ, A. A multicolored set of *in vivo* organelle markers for co-localization studies in Arabidopsis and other plants. *Plant J.* **51,** 1126–1136 (2007).
4. Stougaard, J. *Agrobacterium rhizogenes* as a Vector for Transforming Higher Plants. Application in *Lotus corniculatus* Transformation. *Methods Mol. Biol.* **49,** 49–61 (1995).
5. Spaink, H. P., Wijfjes, A. H. & Lugtenberg, B. J. *Rhizobium* NodI and NodJ Proteins Play a Role in the Efficiency of Secretion of Lipochitin Oligosaccharides. *J. Bacteriol.* **177,** 6276–6281 (1995).
6. Maillet, F. *et al.* Fungal lipochitooligosaccharide symbiotic signals in arbuscular mycorrhiza. *Nature* **469,** 58–63 (2011).
7. Kaneko, T. *et al.* Complete Genome Structure of the Nitrogen-fixing Symbiotic Bacterium *Mesorhizobium loti*. *DNA Res.* **7,** 331–338 (2000).
8. Markmann, K., Giczey, G. & Parniske, M. Functional Adaptation of a Plant Receptor-Kinase Paved the Way for the Evolution of Intracellular Root Symbioses with Bacteria. *PLoS Biol.* **6,** e68 (2008).
9. Sullivan, J. T. & Ronson, C. W. Evolution of rhizobia by acquisition of a 500-kb symbiosis island that integrates into a phe-tRNA gene. *Proc. Natl. Acad. Sci.* **95,** 5145–5149 (1998).
10. Rodpothong, P. *et al.* Nodulation Gene Mutants of *Mesorhizobium loti* R7A—*nodZ* and *nolL* Mutants Have Host-Specific Phenotypes on *Lotus* spp. *Mol. Plant Microbe Interact.* **22,** 1546–1554 (2009).
11. Jarvis, B. D. W., Pankhurst, C. E. & Patel, J. J. *Rhizobium loti*, a New Species of Legume Root Nodule Bacteria. *Int. J. Syst. Bacteriol.* **32,** 378–380 (1982).
12. Tisserant, E. *et al.* Genome of an arbuscular mycorrhizal fungus provides insight into the oldest plant symbiosis. *Proc. Natl. Acad. Sci. USA.* **110,** 20117–20122 (2013).
13. Kosuta, S., Winzer, T. & Parniske, M. in *Lotus japonicus Handbook* (ed. Marquez, A. J.) 87–95 (Springer-Verlag, Dordrecht, The Netherlands, 2005).
14. Hewitt, E. J. , *Sand and Water Culture Methods used in the Study of Plant Nutrition, Tech. Commun.* 22 (Commonwealth Bureau of Horticulture and Plantation Crops, Commonwealth Agricultural Bureaux, Farnham Royal, Buckinghamshire, 1952).
15. Becard, G. & Fortin, J. A. Early events of vesicular-arbuscular mycorrhiza formation on Ri T-DNA transformed roots. *New Phytol.* **108,** 211–218 (1988).
16. Vierheilig, H., Coughlan, A. P., Wyss, U. & Piche, Y. Ink and Vinegar, a Simple Staining Technique for Arbuscular-Mycorrhizal Fungi. *Appl. Environ. Microbiol.* **64,** 5004–5007 (1998).
17. Novero, M. *et al.* Dual requirement of the *LjSym4* gene for mycorrhizal development in epidermal and cortical cells of *Lotus japonicus* roots. *New Phytol.* **154,** 741–749 (2002).
18. Lohmann, G. V. *et al.* Evolution and Regulation of the *Lotus japonicas LysM*

Receptor Gene Family. *Mol. Plant Microbe Interact.* **23,** 510–521 (2010).
19. Volpe, V. *et al.* An AM-induced, *MYB*-family gene of *Lotus japonicas* (*LjMAMI*) affects root growth in an AM-independent manner. *Plant J.* **73,** 442–455 (2013)
20. Imaizumi-Anraku, H. *et al.* Plastid proteins crucial for symbiotic fungal and bacterial entry into plant roots. *Nature* **433,** 527–531 (2005).
21. Buendia, L., Wang, T., Girardin, A & Lefebvre, B. The LysM receptor-like kinase SlLYK10 regulates the arbuscular mycorrhizal symbiosis in tomato. *New Phytol.* **1,** 184–195 (2015).
